# Development of a DNA Metabarcoding Method for the Identification of Crustaceans (*Malacostraca*) and Cephalopods (*Coleoidea*) in Processed Foods

**DOI:** 10.3390/foods14091549

**Published:** 2025-04-28

**Authors:** Julia Andronache, Margit Cichna-Markl, Stefanie Dobrovolny, Rupert Hochegger

**Affiliations:** 1 Department of Molecular Biology and Microbiology, Institute for Food Safety, Austrian Agency for Health and Food Safety (AGES), Spargelfeldstraße 191, 1220 Vienna, Austria; julia.andronache@ages.at (J.A.); stefanie.dobrovolny@ages.at (S.D.); 2 Department of Analytical Chemistry, Faculty of Chemistry, University of Vienna, Währinger Straße 38, 1090 Vienna, Austria; 3 Vienna Doctoral School in Chemistry (DoSChem), University of Vienna, 1090 Vienna, Austria

**Keywords:** seafood, DNA metabarcoding, next-generation sequencing, food authentication, species identification, crustaceans (*Malacostraca*), cephalopods (*Coleoidea*), mitochondrial 16S rDNA

## Abstract

Seafood is a valuable commodity with increasing demand, traded for billions of USD each year. The volatility in supply chains and fluctuating prices contribute to the susceptibility of the seafood market to food fraud. Analytical methods are required to identify seafood in processed foods to ensure food authenticity and compliance with European laws. To address this need, we developed and validated a DNA metabarcoding method for the authentication of crustaceans and cephalopods in processed food samples, as both are prone to food fraud, especially in mixed products. A ~200 bp barcode of the mitochondrial 16S rDNA was selected as the marker for identification and sequenced on Illumina platforms. The DNA metabarcoding method utilizes two primer systems, one for the amplification of crustacean DNA and another for cephalopods. The crustacean primer system comprises two forward and two reverse primers, while the cephalopod primer system includes three forward and one reverse primer. DNA extracts from reference materials, model foods, processed foodstuffs, and DNA extract mixtures were investigated. Even species with a close phylogenetic relationship were successfully identified and differentiated in commercial samples, while single species were detected at amounts as low as 0.003% in model foods. However, false-negative results were obtained for certain species in DNA extract mixtures, which are most likely due to degraded or low-quality DNA and can best be prevented by optimized DNA extraction procedures. Our DNA metabarcoding method demonstrates strong potential as a qualitative screening tool in combination with other in-house DNA metabarcoding methods for food authentication in routine analysis.

## 1. Introduction

Seafood offers a great diversity of food and is composed of healthy nutrients such as protein, omega-3 fatty acids, minerals, and vitamins, supporting a healthy human diet. Therefore, seafood has become a highly traded and an economically significant commodity, with fisheries and aquaculture production peaking at 223.2 million tons, valued at USD 472 billion in 2022 [1].

Discrepancies between supply and demand contribute to pronounced price fluctuations and enhance the vulnerability to food fraud [2]. Food fraud has been defined as the intentional marketing of products that do not conform to consumer expectations for financial gain [3]. However, fraud may also be motivated by more opportunistic factors, such as meeting the requirements of the market by replacing less available species with species of higher availability [3,4]. In the case of seafood, the removal of morphological features during food processing increases the difficulty of species identification [5], while trading networks have grown more intricate due to globalization, creating increased opportunities for fraudulent practices [6].

Regardless of motivation, correct labeling is essential to ensure consumers can make informed choices regarding fair trade, conservation efforts, as well as ethical and religious beliefs [6,7]. For example, crustaceans and mollusks—including bivalves and cephalopods—are not included in kosher diets [8]. Furthermore, seafood allergies, particularly to crustaceans and cephalopods (a subcategory of mollusks), are common and may lead to severe physical reactions [9]. Thus, food fraud involving mislabeling and species substitution poses a potential health risk to consumers.

To regulate food authenticity and food safety, EU Regulation 1169/2011 mandates clear and truthful labeling of food products [10], while Regulation 1379/2013 provides additional requirements for seafood. Fresh or slightly processed seafood products must display a scientific name and a commercial designation on their label, whereas preserved and more processed products may display only a common trade name. Each EU member state must publish a list of accepted commercial designations with corresponding scientific names [11]. In Austria, the *Codex Alimentarius Austriacus* defines these commercial as well as scientific designations and customary seafood [12]. Effective enforcement of these legislations requires validated and standardized analytical methods to detect food fraud and to ensure consumer trust and safety [13].

This study focused on the identification and differentiation of crustaceans and cephalopods, as both prawns [14] and cephalopods have been described as susceptible to food fraud, especially in commercial foods containing both crustaceans and cephalopods [15]. Various methods have been developed to verify seafood authenticity. These include the matrix-assisted laser desorption ionization time-of-flight mass spectrometry (MALDI-TOF MS) approach for the identification of six economically important shrimp species [16] as well as liquid chromatography–mass spectrometry (LC-MS and LC-MS/MS) methods for the identification of fish species [17]. However, the susceptibility of proteins to heat-induced denaturation compromises their suitability for species identification in highly processed foods, rendering DNA-based methods more appropriate for such analyses [7,18,19]. Species-specific real-time polymerase chain reaction (PCR) methods were developed for the identification of *Penaeus monodon*, *Litopenaeus vannamei*, *Fenneropenaeus indicus* [20], and *Sepia officinalis* [21]. This technology is limited in its capacity to detect unknown species, as it requires specific primers and probes for each target [19] and is constrained by the number of detection channels in the PCR instrumentation [22]. In contrast to that, DNA barcoding offers a broader screening capacity by applying universal primers that bind to conserved regions and amplify a variable target region that is called DNA barcode. DNA sequences are obtained via Sanger sequencing and subsequently used for the identification and differentiation of taxa [23]. DNA barcoding has been widely employed for seafood species identification [15,18,24,25,26,27,28,29,30,31,32]; however, it is not applicable for the analysis of complex mixtures containing multiple components due to limitations in interpreting electropherograms [33].

Combining DNA barcoding with next-generation sequencing (NGS) technology, an approach called DNA metabarcoding, enhances efficiency and performance. The primary advantage of DNA metabarcoding is its capacity for untargeted screening [34]. DNA metabarcoding was employed in numerous studies for fish species identification [35,36,37,38,39,40,41,42], and other studies identified both fish and cephalopods in (processed) foods [33,43,44]. Hu et al. recently utilized DNA metabarcoding to identify shrimp species in surimi-based products [45].

The aim of the present study was the development of a DNA metabarcoding method for the identification and differentiation of crustaceans and cephalopods in processed foods, since products such as frozen seafood mixes are often composed of both types of seafood. Mitochondrial DNA (mtDNA) has been more frequently employed for species detection than nuclear DNA because of a higher mutation rate [18] and higher copy numbers per cell [23]. The latter studies targeted the mitochondrial 16S rDNA gene since its conserved regions have demonstrated greater universality than those of the cytochrome oxidase subunit I (COI) or cytochrome b (cytb) genes. In this study, the mitochondrial 16S rDNA was also selected as the target gene due to its short hypervariable, species-specific regions in addition to the highly conserved regions suited for the design of universal primers [46]. The location of the primer binding sites enabled the amplification of short fragment lengths [47], which is critical for compatibility with our established methods for bivalves [19], insects [22], and mammals and poultry [48]. All these methods, designed for application with Illumina sequencing platforms, operate with 300 bp sequencing cartridges. Therefore, we focused on short barcodes (100–300 bp), so-called mini-barcodes, to adapt to potentially degraded DNA in commercial foods [46]. Additionally, barcodes exceeding 300 bp are not suited for paired-end sequencing using 300 bp chemistry. Paired-end sequencing provides more accurate read alignment as well as enhanced detection of base insertions and deletions, making it preferable to single-read sequencing [49].

Although prior studies have presented DNA metabarcoding methods for the identification of crustaceans and cephalopods [43,45,50,51], these methods were not compatible with the specific requirements of our established laboratory workflow—namely, the use of the 16S rDNA marker gene, short barcode lengths suitable for processed food samples (<300 bp), and compatibility with Illumina platforms. Moreover, PCR conditions, including temperature profiles, needed to align with our validated protocols to allow seamless integration into routine analysis. In addition, we originally aimed at combining crustacean and cephalopod primer systems.

To achieve these goals, we aimed not only to expand upon existing methodologies [33,44] but also to design a method that could be harmonized with preexisting assays in our laboratory. As part of this development, we modified primers to increase the universality of the primer systems, thereby enhancing their applicability across a broader range of crustacean and cephalopod species. The method was validated, and a customized database was created to facilitate automated data analysis, enabling its application within an official food control laboratory.

## 2. Materials and Methods

### 2.1. Sample Collection

In total, 171 seafood samples were obtained from local supermarkets, fish markets, delicacy shops, and online stores for use in this study. The samples were either fresh, deep-frozen, or in processed condition. According to the *Codex Alimentarius Austriacus*, the term “fresh” refers to an untreated product that is merely cleaned, gutted, cut, and chilled [12]. All products were sourced commercially; therefore, no ethical concerns were anticipated. DNA was extracted from 65 samples and analyzed via Sanger sequencing (Microsynth, Balgach, Switzerland) to verify species identity. The obtained sequences were compared to public databases provided by the National Center for Biotechnology Information (NCBI, Bethesda, MD, USA [52]). The remaining 106 samples were classified as processed food samples. Canned samples were stored at room temperature, and other samples were stored at −20 °C until DNA extraction. Sample selection criteria included the representation of a broad range of species to investigate the applicability of the selected barcode, and the commercial and legal relevance of the species listed in the *Codex Alimentarius Austriacus*. The *Codex Alimentarius Austriacus* currently includes 54 species, 29 genera, and 4 families of crustaceans and 16 species and 8 genera of cephalopods [12]. Among these, our reference material covered 14 species, 6 genera, and 4 families of crustaceans along with 8 species and 4 genera of cephalopods. In certain cases, individual samples represented multiple taxonomic levels listed in the *Codex Alimentarius Austriacus* such as *Cancer pagurus*, which corresponds to the genus *Cancer* spp. and the family *Cancridae*.

### 2.2. DNA Extraction with CTAB Protocol and Quantification

DNA extraction was performed using a hexadecyltrimethylammonium bromide (CTAB) buffer-based isolation method, adapted from Dobrovolny et al. [48]. Initially, samples were homogenized in a lab mill, and 1 g of homogenate was mixed with 7 mL of a CTAB extraction buffer (2% (*w*/*v*) CTAB (VWR International, Radnor, PA, USA), 0.1 M tris(hydroxymethyl)aminomethane (Tris; VWR International, Radnor, PA, USA), 0.02 M ethylenediaminetetraacetic acid disodium salt dihydrate (EDTA; Merck, Darmstadt, Germany), and 1.4 M sodium chloride (Merck, Darmstadt, Germany)) adjusted to pH 8.0 with 4 M hydrochloric acid (Merck, Darmstadt, Germany). After 80 µL proteinase K solution was added (600 mAnson-U/mL; Merck, Darmstadt, Germany), the mixtures were incubated overnight at 56 °C and 195 rpm (Certomat BS 1, B. Braun Biotech International, Berlin, Germany). In the course of this study, the predigestion step was optimized to account for the protein content of seafood; details are provided in Section 3.3. Following incubation, samples were centrifuged for 10 min at 3900× *g* (Centrifuge 5810R, Eppendorf AG, Hamburg, Germany), followed by transferring 1 mL of the supernatant to a 2 mL Eppendorf tube containing 600 µL chloroform/isoamylalcohol (24:1 (*v*/*v*), Sigma-Aldrich, St. Louis, MO, USA; Merck, Darmstadt, Germany/VWR International, Radnor, PA, USA). After vortexing for 30 s, the mixture was centrifuged at 20,000× *g* for 10 min (Centrifuge 5424R, Eppendorf AG, Hamburg, Germany). A total of 300 µL of the aqueous phase was transferred to a new 2 mL Eppendorf tube and mixed with 300 µL lysis buffer (Promega, Madison, WI, USA), and 5 µL of RNase A (4 mg/mL; Promega, Madison, WI, USA) was added. The tube was incubated in a thermomixer (Thermomixer C, Eppendorf AG, Hamburg, Germany) at 65 °C for 15 min at 1000 rpm. After cooling to room temperature, samples were centrifuged. DNA was isolated using the Maxwell RSC Pure-Food GMO and Authentication Kit (Promega, Madison, WI, USA), following the manufacturer’s instructions.

DNA concentrations were determined fluorometrically (Qubit^®^ 2.0 fluorometer, Thermo Fisher Scientific, Waltham, MA, USA). The Qubit^®^ dsDNA broad-range assay kit (2 to 1000 ng) was used for higher concentrations, and for lower concentrations, the Qubit^®^ dsDNA high-sensitivity assay kit (0.2 to 100 ng) was used. DNA purity was assessed from the ratio of the absorbance at 260 nm/280 nm and 260 nm/230 nm (QIAxpert spectrophotometer, software version 2.2.0.21, Qiagen, Hilden, Germany). The DNA extraction procedure was checked for cross-contamination through the inclusion of negative extraction controls. DNA extracts were stored at 4 °C.

### 2.3. Preparation of DNA Extract Mixtures

DNA extract mixtures were prepared for method validation. First, individual DNA extracts were diluted to a concentration of 5 ng/µL. Mixtures were designed to reflect taxonomic relationships, incorporating species from either the same family or from different families. Additionally, varying ratios (% *w*/*w*) were used to assess detection across a range of concentrations. Certain mixtures included species from other taxonomic classes as the major components, such as pig (*Sus scrofa*), chicken (*Gallus gallus*), fish (*Sparus aurata*, *Gadus chalcogrammus*), corn (*Zea mays*), and mussel (*Mytilus* spp.). In total, 34 mixtures were prepared and analyzed.

### 2.4. Reference Sequences, DNA Barcodes, and Primers

Reference sequences for seafood species commercially available in Austria, as listed in the *Codex Alimentarius Austriacus* [12], were downloaded from the NCBI databases [52] (Supplementary Table S1). Complete reference sequences of the mitochondrial genome were preferred due to their higher reliability. In cases where complete sequences were unavailable, entries containing the full barcode region without unidentified nucleotides were selected for the customized database.

FASTA files were edited using the CLC Genomics Workbench software (version 10.1.1, Qiagen, Hilden, Germany). Target barcode regions were extracted, aligned, and compared to assess sequence variation. Default software settings were used for alignment generation, and the alignments were applied for primer design. The primers, adapted from Deagle et al. [53] for crustaceans and from Chapela et al. [54] for cephalopods, amplify a barcode region of the mitochondrial 16S rDNA gene, resulting in average barcode lengths of 210 bp for crustaceans and 197 bp for cephalopods. Two forward and two reverse primers were designed for crustaceans, while three forward primers and one reverse primer were designed for cephalopods (Table 1).

**Table 1:** Primer sequences evaluated in this study compared to previously published primers [53,54]. Primers designated “K” target crustaceans, while “W” primers target cephalopods. Mismatches to respective published primers are highlighted in red; missing bases at the 5′ or 3′ ends are highlighted in green. Wobble positions are indicated in blue.

Name	Sequence 5′ → 3′
Fwd crustaceans [53]	GACGAKAAGACCCTA
FwdK-1	GGGGACGATAAGACCCTATAAA
FwdK-2	AAAGACGATAAGACCCTATAAA
Fwd cephalopods [54]	GACGAGAAGACCCTAATGAGCTTT
FwdW-1	GGACGAGAAGACCCTATTGAG
FwdW-2	GGACGAGAAGACCCTAATGAG
FwdW-3	GGACGAAAAGACCCTATTGAG
Rev crustaceans [53]	CGCTGTTATCCCTADRGTAACT
RevK-1	ATTACGCTGTTATCCCTAAAGTA
RevK-2	ATAACGCTGTTATCCCTAAAGTA
Rev cephalopods [54]	CAAATTACGCTGTTATCCCTATGG
RevW-1	ACGCTGTTATCCCTATGGTAA
**Illumina Overhang Adapter Sequences**	
Forward	TCGTCGGCAGCGTCAGATGTGTATAAGAGACAG
Reverse	GTCTCGTGGGCTCGGAGATGTGTATAAGAGACAG

Primer dimerization potential (Gibbs free energy), annealing temperatures, and melting temperatures were assessed using Oligo Calc, the OligoAnalyzer Tool provided by Integrated DNA Technologies (IDT, Coralville, IA, USA), and online product descriptions from TIB Molbiol (Berlin, Germany). The primers, including overhang adapter sequences, were obtained from TIB Molbiol.

Primer efficiency was evaluated via real-time PCR using DNA extracted from individual reference samples. Previously published PCR conditions were applied, including 12.5 ng DNA input and the ‘ready-to-use’ HotStarTaq Master Mix Kit from Qiagen (Hilden, Germany) at a 1× final concentration [48]. Magnesium chloride concentration and primer concentrations were optimized. Real-time PCR was performed using a fluorescent intercalating dye (EvaGreen^®^ (20× in water)) in 96-well plates on the LightCycler^®^ 480 System (Roche, Penzberg, Germany). Each 25 µL PCR reaction comprised 22.5 µL reaction mix and 2.5 µL DNA extract. The no-template control (NTC) contained 2.5 µL water. The specificity of the PCR products was evaluated using agarose gel electrophoresis and melting curve analysis.

### 2.5. Library Preparation and NGS

PCR products were sequenced on the MiSeq^®^ and iSeq^®^ 100 platforms (Illumina, San Diego, CA, USA). Prior to library preparation, DNA extracts were diluted to 5 ng/µL. DNA extracts with concentrations below 5 ng/µL were used undiluted. DNA library preparation followed the protocol by Dobrovolny et al. [48] with modifications as described by Hillinger et al. [22], including the use of 36 µL magnetic beads and an average library size of 197 bp or 210 bp. For sequencing on MiSeq^®^, libraries were diluted to 4 nM with 10 mM Tris-HCl (pH 8.6), and 5 µL of each library was pooled. For sequencing on iSeq^®^ 100, libraries were diluted to 1 nM, and 7 µL of each library was pooled. DNA library pool concentrations were determined with the Qubit^®^ 4.0 fluorometer (Thermo Fisher Scientific, Waltham, MA, USA). Final loading concentrations were 8 pM with 5% PhiX spike (MiSeq^®^) and 30 pM with 5% PhiX spike (iSeq^®^ 100) for all sequencing runs. DNA denaturation was performed using 0.2 M NaOH prior to loading on MiSeq^®^, while denaturation occurred within the cartridge after loading on the iSeq^®^ 100 platform. Sequencing was conducted using the MiSeq^®^ Reagent Kit v2 (300 cycles) or the iSeq^®^ 100 i1 Reagent Kit v2 (300 cycles).

Reference samples were sequenced in one or two replicates across one or two sequencing runs on at least one of the sequencing platforms and compared individually to reference sequences. Commercial food products were sequenced using either platform. DNA extract mixtures were sequenced in two or four replicates across two sequencing runs on the MiSeq^®^ platform.

### 2.6. NGS Data Analysis with Galaxy

Raw sequencing image data (bcl-files) were processed using Illumina’s bcl2fastq2 conversion software (version 2.19.0.316, Illumina, San Diego, CA, USA) to generate FastQ files. Downstream analysis was conducted using a modified version of a published pipeline in Galaxy (version 19.01) [55]. Adapter and primer sequences were removed prior to merging of paired-end reads. Dereplicated sequences were aligned against a customized database of pre-allocated reference sequences from NCBI, using the Basic Local Alignment Search Tool (BLASTn [56]) for taxonomic assignments [57]. The percent identity cutoff was set to 97%.

The current customized database includes crustaceans assigned to the class *Malacostraca* and cephalopods assigned to the subclass *Coleoidea* (Supplementary Table S1). An exception from *Malacostraca* is represented by *Pollicipes* spp. (class *Theocostraca*).

## 3. Results and Discussion

This study aimed to develop and validate a DNA metabarcoding method for the identification and differentiation of crustaceans and cephalopods in processed foods. Validation parameters included repeatability, selectivity, cutoff value, and robustness. The universal primers were also evaluated for their coverage across taxa. In total, 178 samples were analyzed, including 65 reference samples, 106 commercial food samples, 7 model foods as proficiency test samples, and an additional set of 34 DNA extract mixtures.

### 3.1. In Silico Alignment Studies of the 16S rDNA Barcodes

**Figure 1:**
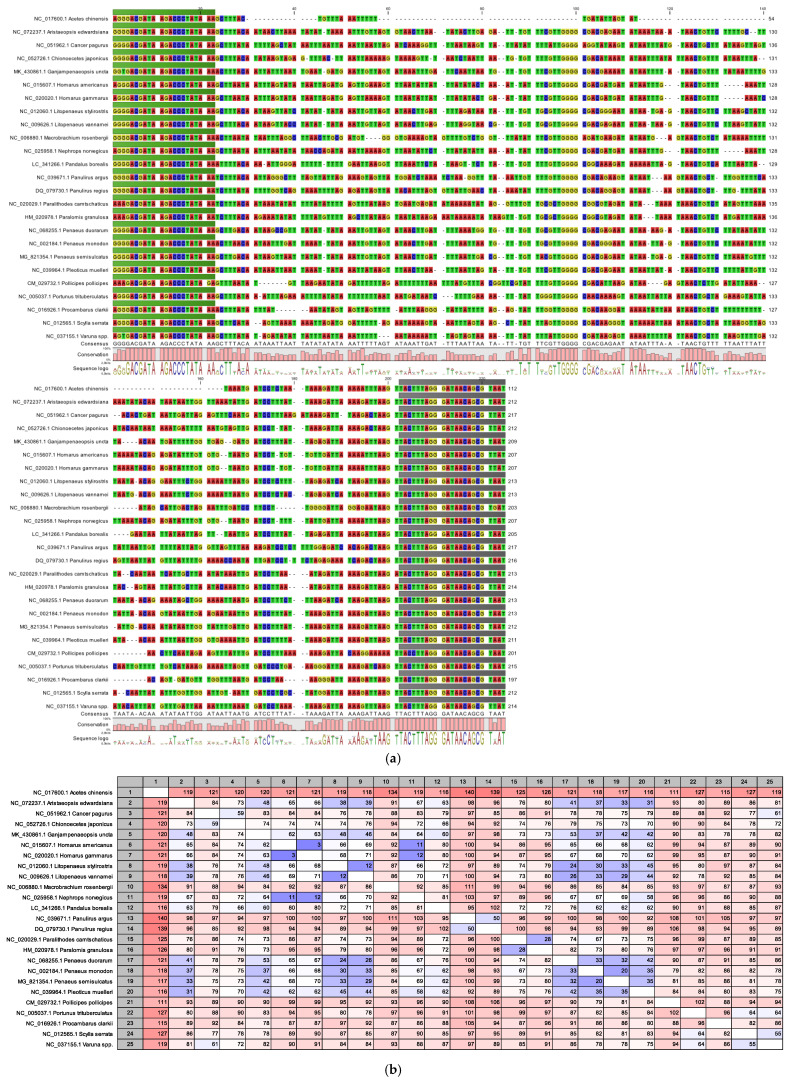

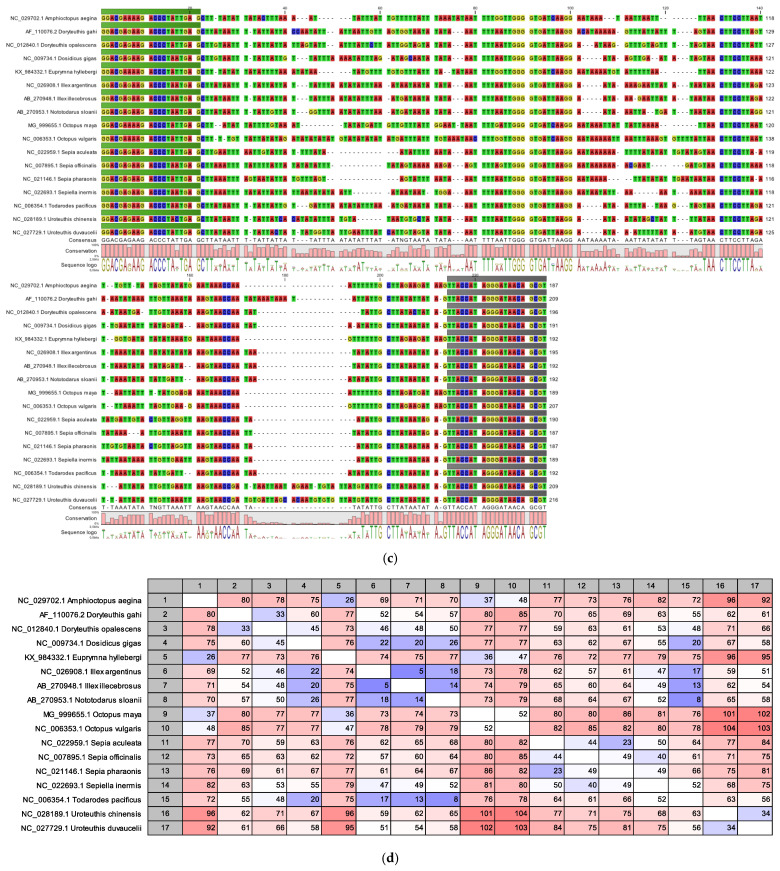
Alignments (**a**,**c**) and pairwise alignment comparisons (**b**,**d**) of mitochondrial 16S rDNA barcodes for crustacean (**a**,**b**) and cephalopod (**c**,**d**) reference species analyzed in this study. Universal primer binding sites are indicated in green (forward primer) and gray (reverse primer). Primer binding sites were removed prior to alignment comparison to prevent bias in barcode variability assessment. Digits indicate pairwise differences between compared sequences, representing varying nucleotides, while colors indicate the degree of nucleotide differences, ranging from blue (few differences) to red (many differences) (CLC Genomics Workbench software).

Prior to inclusion into the customized database, DNA barcodes were aligned and compared using the CLC Genomics Workbench software. The alignments (crustaceans 1a, cephalopods 2a) and the alignment comparisons (crustaceans 1b, cephalopods 2b) in Figure 1 illustrate the mitochondrial 16S rDNA barcodes from reference species. Primer binding sites were removed prior to alignment comparison to prevent site-specific variabilities from influencing the number of different bases between barcodes, indicated by digits in the alignment comparisons.

For some samples, such as *Penaeus monodon*, the most abundant dereplicated sequence obtained from the sample did not exhibit complete identity with the corresponding reference sequence. To resolve this, sequences were subjected to BLASTn analysis against the NCBI database to determine the closest taxonomic match. The barcode sequence exhibiting the greatest overlap with the sequencing result was incorporated into the customized database as an additional reference. In instances where the sequence matched multiple species equally, it was added under the designation of the Lowest Common Ancestor (LCA) [58].

In silico analysis indicated that differentiation of all taxa listed in the *Codex Alimentarius Austriacus* can be accomplished, except for 15 crustacean taxa and 2 cephalopod species. For 15 of these taxa, either no 16S rDNA reference sequences were available in the NCBI database or the sequences did not contain the full barcode region. The two remaining species could not be identified on the species level, despite being listed as species in the *Codex Alimentarius Austriacus*.

The customized database for crustaceans comprises 350 barcodes, of which 94.6% enable identification on the species level, 4.8% on the genus level, and 0.6% above the genus level. The customized database for cephalopods contains 105 barcodes, of which 92.4% enable identification on the species level, 6.7% on the genus level, and 0.9% on higher taxonomic levels. The greater number of crustacean entries reflects the broader regulatory coverage of this group, with 87 entries in the *Codex Alimentarius Austriacus* compared to 24 entries for cephalopods.

Barcodes identical across different taxa were assigned to the LCA and included in the database; for example, *Octopus conispadiceus*:*Enteroctopus dofleini* demonstrated an identical barcode (LCA: *Octopodoidea*). LCA assignment indicates that species identification is not feasible, which is critical in official control laboratories, as results could be subject to legal debate. Asserting a higher taxonomic resolution than the method supports may lead to regulatory implications. Barcodes identical among species within the same genus were included under the corresponding genus name. These species are displayed in Table 2.

### 3.2. Development of Primer Systems, PCR Assays, and Analysis of Reference Material with DNA Metabarcoding

Originally, one aim was to develop a duplex assay for simultaneous amplification of crustacean and cephalopod DNA, as commercial foods such as frozen seafood mixes often contain both types of seafood. However, this primer duplex did not have the capacity to distinguish between different species of both *Coleoidea* and *Malacostraca*. The cephalopod reverse primer cross-reacted with crustacean DNA, resulting in preferential amplification and consequently amplification bias in favor of crustaceans. This hindered cephalopod detection in mixed samples. Therefore, the duplex assay was divided into two singleplex assays, enabling the amplification and detection of both crustacean and cephalopod species.

During method development, primers were designed and parameters for the amplicon PCR were established with the objective of combining the new method with other published in-house methods [19,22,48] to streamline various assays for routine application. The selected primer binding sites (Figure 1) have been previously characterized as highly conserved within crustaceans and cephalopods. However, conservation across taxonomic classes remains limited [53,59]. Similar primers have previously been applied for crustacean identification, but not in the context of food authentication [53]. For cephalopods, similar primers were used in the analysis of surimi products [33].

Primers from the literature [53,54] were adapted, to better align with the conserved regions, and evaluated using DNA extracts from various species. The primers successfully amplified the DNA templates, without the formation of unspecific side products. PCR products had the expected fragment length, determined by gel electrophoresis. Different PCR parameters were examined in preliminary tests such as primer concentrations ranging from 0.2 to 0.8 µM in the final reaction mix. A touchdown PCR was performed to determine the optimal annealing temperature, with annealing temperatures varying from 65 °C to 62 °C, 62 °C to 59 °C, and 59 °C to 56 °C in three different amplification programs. Afterwards, annealing temperatures of 58 °C and 62 °C were evaluated to determine, whether the primer systems could be combined with other in-house methods. At 58 °C, stronger gel bands were obtained, indicating stronger amplification, so it was selected for the final amplicon PCR protocol.

After initial sequencing analyses of reference samples, food products, and DNA extract mixtures, preferential amplification of certain species was observed. To optimize species detection, primers for crustaceans and cephalopods were redesigned. An additional forward primer was added to each assay, and the reverse primers for crustaceans were rearranged. For this purpose, mismatches in the reverse primer were shifted towards the 5′ end to increase primer affinity, since mismatches near the 3′ end have a greater influence on amplification efficiency [60]. Primers were evaluated initially in singleplexes, then in class-specific multiplexes.

To improve the detectability of certain species in mixtures, mismatch-free primers targeting those species were tested at increased concentrations. Initially, forward primers were applied at 0.4 µM and reverse primer concentrations were adapted accordingly: 0.4 µM for crustaceans and 1.2 µM for cephalopods. To assess the effect of altered primer ratios, the concentration of selected primers was increased from 0.4 µM to 0.6 µM, yielding a 3:2 ratio. However, amplification efficiency was not improved. As a result, original primer concentrations were maintained.

The addition of 1.5 mM or 3 mM magnesium chloride was evaluated (final concentration of magnesium chloride: 3 mM or 4.5 mM). Slightly lower C_t_ values with an average decrease of 1.4 were obtained across 22 reference crustacean species with the addition of 1.5 mM magnesium chloride. For *Aristaeopsis edwardsiana*, no fluorescence signal was detected regardless of magnesium chloride addition. In contrast, *Pollicipes pollicipes* and *Varuna* spp. yielded C_t_ values of 30 with additional magnesium chloride, whereas no fluorescence signal was observed previously. Magnesium chloride was not added to the cephalopod reaction mix, as it showed no impact on amplification in real-time PCR.

PCR cycling conditions remained identical for both primer systems: initial denaturation at 95 °C for 15 min; 35 cycles at 95 °C, 58 °C, and 72 °C for 30 s each; and a final elongation at 72 °C for 10 min. The amplicon PCR conditions correspond to those used in our DNA metabarcoding protocol for insect amplification [22], allowing parallel amplification of insects, crustaceans, and cephalopods.

The final DNA metabarcoding method was applied to individual DNA extracts from reference samples. The results of this analysis are summarized in Table 3, including the total number of raw reads, the total number of reads that passed the analysis pipeline in Galaxy, and the number of correctly assigned reads based on one or two replicates. Sanger sequencing results were confirmed; however, in some samples, both methods identified a different species than expected based on product labeling. Discrepancies between declared and identified species were observed for several samples (sample 13, 14, 24, 29, 30, 35, 46–48, 57). These were retained as reference samples due to their relevance in validation mixtures, according to the literature (Section 3.3). With the exception of sample 3, >75% of raw reads passed the workflow of the Galaxy pipeline. Correctly assigned reads ranged from >15,000 to >127,000, allowing reliable species identification of crustaceans and cephalopods. Sample 64 could only be identified on the genus level due to identical barcodes among different species.

Sample 65 contained both *Homarus gammarus* and *Homarus americanus*. As both species belong to the same genus, the presence of *Homarus americanus* DNA was regarded as a minor contamination, and the DNA extract was retained for use in validation mixtures.

### 3.3. Analysis of DNA Extract Mixtures

Primer selectivity was evaluated using BLASTn analysis against the NCBI database to identify taxa with the same primer binding sites as *Malacostraca* and *Cephalopoda*. *Malacostraca* and *Cephalopoda,* as well as models and uncultured/environmental sample sequences, were excluded from the search. The accordance between crustacean primer sequences and BLASTn search results ranged from 95 to 100% (≤1 mismatch), and from 80 to 100% (≤4 mismatches) for cephalopod primer sequences. Real-time PCR followed by gel electrophoresis was performed on selected species available in our sample stock that represented classes which matched BLASTn hits. Amplification was observed for four insect species applying the crustacean primers, and for a marine gastropod and a locust using the cephalopod primers. All amplicons demonstrated the expected fragment length. These results align with Lorusso et al. [59], who reported that insect DNA could be amplified using 16S rDNA primers designed for crustaceans and cephalopods via in silico analysis.

Detectability varied among species in preliminary tests conducted prior to method validation. In silico evaluations were performed using The ViennaRNA Web Services [61] to assess primer binding site accessibility and the potential formation of secondary structures such as hairpins. The formation of secondary structures in the single-stranded DNA template may affect amplification efficiency due to competition between intermolecular primer binding and intramolecular template hybridization [62]. Comparable secondary structures were predicted for the DNA templates of *Dosidicus gigas* and *Illex illecebrosus* (Supplementary Figure S1). However, the detectability of both species varied considerably in validation mixtures (Table 4). These findings suggest that secondary structure formation was not the primary factor contributing to false-negative results.

Several strategies were implemented to improve the detection of species previously yielding false-negative results. Due to the high protein content of seafood [63], DNA extraction was modified through the addition of Collagenase D (5 µL of a 270 MandelU/mL solution) and 3 mL of water to the homogenate prior to sample treatment with Proteinase K. This predigestion was performed at 37 °C and 195 rpm for 1 h. An additional modification included increasing the amount of sample from 1 g to 2 g, as well as using 160 µL of Proteinase K instead of 80 µL and 10 mL CTAB buffer instead of 7 mL. Further tests involved redoubling the DNA concentration in the PCR reaction mix from 5 ng/µL to 10 ng/µL and adjusting the sequencing depth to improve the detection of components present at low concentrations.

As the optimized extraction protocols did not improve detectability, and DNA extracts obtained via the original extraction protocol (Section 2.2) showed sufficient DNA purities, the initial extraction protocol was retained. DNA extracts in validation mixtures were also substituted with alternative extracts from different reference samples of the same species. Extracts exhibiting the most pronounced slope of the amplification curve and the highest melting peaks were used, leading to improved detection for certain species. This indicates that DNA integrity may have been compromised in certain samples. However, for some species, detectability remained insufficient, and the underlying cause remains unresolved.

DNA extract mixtures were prepared to characterize the repeatability, selectivity, cutoff value, and robustness as part of method validation. The composition of the mixtures and the results are displayed in Table 4. Mixtures containing minor components <5% were analyzed in duplicates across two sequencing runs, and mixtures containing minor components ≥5% were analyzed in single replicates across two sequencing runs. Total read counts ranged from 79,723 to 157,230, with 78,069 to 152,906 reads passing the Galaxy pipeline. Comparable proportions of species-assigned reads across sequencing runs indicated the method’s repeatability.

To simulate worst-case scenarios, ternary DNA mixtures (mixtures 1–3, 16–18; Table 4) were prepared by combining two commercially relevant species exhibiting low threshold cycle (C_t_) values, ranging from 17 to 24 for crustaceans and 18 to 25 for cephalopods, with a third species as the minor component. This evaluation aimed to assess the possibility of false-negative results. In general, an underestimation of certain species, including false-negative results, was observed, with the exception of the main component. *Panulirus argus* was the only overestimated species in mixtures 1–3, which may be due to more efficient amplification, as indicated by a C_t_ difference of 5.3 compared to the main component in single-species samples.

Species with varying C_t_ values were combined to ascertain the accuracy of read assignment. Crustacean mixtures included *Panulirus argus*, *Homarus gammarus,* and *Cancer pagurus* (C_t_: 17.3, 20.5, and 28.0, respectively). The cephalopod mixtures were composed of *Octopus vulgaris*, *Illex argentinus,* and *Uroteuthis duvaucelii* (C_t_: 19.8, 23.9, and 26.7, respectively). The components were subjected to a rotational process, and consequently each species served once as the major component and twice as a minor component (mixtures 8–10 and 23–25, Table 4). Components with the highest C_t_ values were not detected or were strongly underestimated. Species with the lowest C_t_ values were detected and overestimated, regardless of their actual proportion. These findings indicate that quantitative species estimation in mixtures is affected by amplification bias, as is reflected by the C_t_ values of the single-species reference samples. Additionally, high-abundance species may suppress the amplification of minor components. Parameters such as the accessibility of primer binding sites or impaired DNA integrity could also affect species detectability in mixtures; however, these explanations were excluded as contributing factors for certain species based on our results. Primer template mismatches remain a likely explanation for varying amplification efficiency [46], particularly when located near the 3′-end [60]. However, no clear correlation could be established between the number of mismatches and increased C_t_ values in this study. For instance, only *Homarus gammarus* had a single mismatch at the 5′-end of the forward primer in mixtures 8–10, while *Cancer pagurus* did not demonstrate any mismatches. However, *Homarus gammarus* demonstrated stronger detectability compared to *Cancer pagurus*.

In complex mixtures, the proportion of reads assigned to each species does not accurately reflect the actual species proportions. This discrepancy appears to be influenced both by the true abundance of each species and by the specific composition of the mixture.

To evaluate the influence of genetic distance on species detection [64], DNA extract mixtures were prepared containing either closely or distantly related species. Taxonomic relatedness was assessed based on whether species belonged to the same family. Mixtures either contained equal amounts of all species (mixtures 4, 5, 19, and 20; Table 4), or one main species with others in minor amounts (mixtures 6, 7, 21, and 22; Table 4). No correlation was observed between genetic distance and species detectability. In each mixture, one species was overestimated with respect to assigned reads, while the other species were underestimated.

Additional mixtures were composed of pork (*Sus scrofa*), chicken (*Gallus gallus*), fish (*Sparus aurata*, *Gadus chalcogrammus*), corn (*Zea mays*), or mussel (*Mytilus* spp.) as the main ingredients (mixtures 11–15, 30–34, Table 4). These species would, if used in a case of adulteration, represent a common and value-reducing substitution ingredient. Pork and chicken meat have been reported as adulterants in shrimp and squid balls [65], while cephalopods were mixed with *Gadus chalcogrammus* in surimi products [33]. The presence of *Nemipterus* spp. was detected in crab balls, crab legs, and lobster balls [51]. Since the sample stock did not include *Nemipterus* spp., *Sparus aurata* was used in mixture 13. Mixtures containing mussel as the main component were prepared (mixture 15, 34; Table 4) because frozen seafood mixes often comprise mussels as a component. In mixtures 11–14, 30, 33, and 34, no reads were obtained for the main component. A maximum of 2211 reads were obtained for *Sus scrofa* in mixture 31, corresponding to 2.1% of the assigned reads. However, the mixture contained 70% of pork DNA. This lack of cross-reactivity is useful if the method is employed for the selective detection of crustaceans or cephalopods, while screening for other taxa is beneficial for identifying economically motivated adulteration (EMA).

While Lorusso et al. reported that most 16S rDNA primer pairs utilized in their in silico study could amplify and thus detect *Bos taurus*, *Sus scrofa.* and *Gallus gallus*, our primers exhibited higher selectivity. However, one primer pair by Lorusso et al., which exhibited the greatest similarity to our primers with respect to length and the absence of wobble bases, also demonstrated enhanced selectivity [59].

Intra-genera substitution was investigated for *Sepia* spp., *Sepiella* spp., and *Uroteuthis* spp. (mixtures 27–29, Table 4) [15]. Intra-class species substitution involving *Penaeus monodon* and *Litopenaeus vannamei* (mixtures 1–4, 6, and 7, Table 4) [66] as well as *Doryteuthis gahi* with *Illex argentinus* and *Nototodarus sloanii* (mixture 26, Table 4) [67] was also investigated. All species were detected in the respective mixtures, except *Uroteuthis duvaucelii* in mixture 27 and *Sepia officinalis* in mixture 29, demonstrating the method’s capacity to identify these types of counterfeits.

To distinguish species with low read assignments from background noise, a cutoff was calculated from the impurities with the highest number of reads in our validation mixtures. The cutoff amounted to 0.03% for crustaceans and to 0.07% for cephalopods. These findings are consistent with results from Dobrovolny et al. [48], who reported false-positive results below 0.05% of the total reads. However, certain species fell below the cutoff despite being part of the mixtures: *Cancer pagurus* and non-crustacean species for the crustacean primer system as well as *Illex illecebrosus*, *Illex argentinus*, *Sepia officinalis*, *Uroteuthis duvaucelii*, *Gallus gallus*, *Zea mays,* and *Mytilus* spp. for the cephalopod system.

The robustness of this method was evaluated using different Illumina sequencing platforms in combination with different flow cells for MiSeq^®^ (1, 4, or 15 million reads) or iSeq^®^ 100, respectively. Data quality was determined by average Q-Scores (Q30: 1 error base in 1000 bases), clusters passing filter (CPF), and cluster density. On iSeq^®^ 100, Q30 amounted to 92.56%, CPF was 47.35%, and cluster density was 327 K/mm^2^. Of the total reads, 5.79% (percent aligned) were identified as PhiX, with an error rate of 0.63%. On MiSeq^®^, Q30 was 95.02%, CPF amounted to 95.67% with a cluster density of 782 K/mm^2^, and 3.30% PhiX were identified with an error rate of 0.52%. The method runs on both Illumina platforms and delivers high-quality sequencing data.

### 3.4. Analysis of DNA Extracts from Model Foods

To evaluate the accuracy of the method, DNA was extracted and analyzed from model food samples included in two proficiency tests aimed at detecting food allergens in either spice crackers or a potato powder matrix (Table 5). Samples 1–3 were analyzed in the first test, and samples 4–7 were included in the second proficiency test. Sample 3, a spiked sample, consisted of a different matrix than samples 1 and 2. Species were correctly identified in all model food samples at concentrations ranging from 0.003% to 0.015% (% *w*/*w*), corresponding to spike levels of 30.8 mg/kg to 151 mg/kg (Table 5). These results demonstrate the applicability of the DNA metabarcoding method for identifying crustacean species in processed foods.

### 3.5. Analysis of Commercial Processed Foods

To assess the applicability of the DNA metabarcoding method, 106 commercial food samples were analyzed. A variety of food matrices was investigated, such as dried shrimp, fried calamari, frozen seafood mixes, canned products in brine, oil, or sauces, spice blends for instant noodles, prawn crackers, squid ink linguine, pesto, and butter. According to labels, 70 samples contained crustaceans, 31 samples contained cephalopods, and 5 seafood mixes contained both. The results of the sequencing analyses are displayed in Table 6.

Crustaceans and/or cephalopods were identified in all samples except for a soup cube (sample 11). Only short reads—approximately 70 bp—were obtained for this sample, resulting in the inability to match paired-end reads (fastqjoin). The short reads indicated that processing conditions resulted in DNA degradation, as temperature, low pH (~pH 3), high-pressure treatment, drying, and mechanical processing have a negative impact on DNA integrity [46,68]. Similar results were obtained for sample 32, as most of the reads demonstrated a fragment length of approximately 45 bp.

*Litopenaeus vannamei* was the most frequently identified crustacean species, accounting for 46% of all processed food samples containing crustaceans, though it was not invariably the only species present. In 66% of these cases, *Litopenaeus vannamei* was correctly labeled; an additional 16% applied a less specific commercial designation such as “shrimp”, which is in accordance with EU Regulation 1379/2013. The remaining samples were regarded as mislabeled.

No crustacean species was detected in sample 8 despite labeling. In contrast, *Sepia officinalis*, which exhibited low detection rates in DNA extract mixtures, was unambiguously identified in samples 14 and 19 (Table 6). Similarly, *Uroteuthis duvaucelii* was detected in conjunction with *Uroteuthis chinensis* in samples 73, 79 and 81, which were previously difficult to co-detect in DNA extract mixtures. The findings indicated a significant proportion of *Sepia officinalis* and *Uroteuthis duvaucelii* in these samples. Species were detected in processed foods in amounts as low as 0.15% for crustaceans (samples 29 and 30, Table 5) and 0.86% (*w*/*w*) (sample 19) for cephalopods according to declarations.

As observed in DNA extract mixtures, mussels contained in frozen seafood mixes (samples 6–9, 15) were not detected. Instead, rice weevil (*Sitophilus oryzae*) was detected in sample 20 (spaghetti with squid ink). Rice weevil is a pest of cereal grains [69], whose presence likely results from contamination during pasta production. This confirms that the cephalopod primers can amplify insect DNA, which is consistent with BLASTn analysis of our primer sequences and findings by Lorusso et al. [59], who reported cross-amplification of insect DNA by 16S rDNA primers targeting crustaceans and cephalopods.

Of the 106 commercial samples, 81 were labeled at the species or genus level, while 25 samples used broader taxonomic or commercial designations. A total of 33 samples (samples 12, 13, 26, 53, 67, 68, 74, 75, 77–79, 81–92, 94–98, 100–103, 106) were mislabeled. In 11 of these cases, the genus was labeled correctly. An additional 12 samples (samples 6, 9, 14, 15, 37, 66, 69, 70, 72, 73, 76, 80) contained both the labeled species and additional undeclared taxa. This may result from either intentional fraud or unintentional errors along the supply chain, as morphological similarities between species can hinder correct identification.

In accordance with prior studies, several samples contained different or additional species of *Uroteuthis* spp. (samples 6, 9, 15, 66, 72, 73, 79–81) [15], and *Penaeus monodon* was replaced with *Litopenaeus vannamei* (samples 53 and 96) [66]. Multiple *Sepia* species were detected in samples 14, 15, 19, and 20, as previously reported [15]. All samples labeled to contain crustaceans or cephalopods contained taxa from the labeled class, except sample 77. In this case, most reads were assigned to *Loligo vulgaris* (cephalopod) instead of *Crangon crangon* (crustacean). A second analysis confirmed the result, ruling out library preparation errors. No vulnerable or endangered species, as defined by the International Union for Conservation of Nature (IUCN), were identified in processed food samples [70].

## 4. Conclusions

We developed two primer systems suitable for DNA metabarcoding to detect and identify crustaceans and cephalopods in processed foods. The crustacean system comprises two forward and two reverse primers; for the cephalopod system, we utilized three forward and one reverse primer. Compared to real-time and species-specific PCR, this method offers enhanced screening capabilities due to the high conservation of the primer binding sites. Mitochondrial 16S rDNA markers were selected, yielding barcodes of approximately 210 bp for crustaceans and 197 bp for cephalopods. A total of 178 samples were analyzed, including 65 reference samples, 106 commercial food samples, 7 proficiency test model food samples, and 34 DNA extract mixtures. Species were correctly identified in all reference and proficiency test samples. Diverse species were detected in both mixtures and processed foods; notably, 18 processed products contained three or more crustacean and/or cephalopod species. A wide variety of food matrices was analyzed in terms of composition and degree of processing. The method enabled detection in commercial foods at levels as low as 0.15% for crustaceans (0.003% in proficiency test samples) and 0.86% for cephalopods. These findings suggest potential suitability of the DNA metabarcoding method for allergen detection. The method also identified rice weevil DNA in a commercial sample, demonstrating that the cephalopod primer system can amplify insect DNA. Species- or genus-level discrimination for most taxa listed in the *Codex Alimentarius Austriacus* was confirmed through in silico and wet lab analyses, using customized databases that currently include 455 reference sequences for crustaceans and cephalopods. This method is compatible with existing metabarcoding systems for bivalves [19], insects [22], as well as mammals and poultry [48], supporting streamlined and standardized library preparation for routine analysis and potential application in official food control programs.

However, this method is subject to limitations as indicated by the validation results. In mixtures, species detectable in single-species samples were sometimes undetectable at concentrations between 0.5% and 20%, depending on the mixtures’ composition and the relative abundance of each species. Our findings suggest that the observed false-negatives likely resulted from degraded or low-quality DNA. Consequently, the absence of a declared species in a food product does not definitively indicate its absence from the product, as previously noted by Ballin et al. [7]. This has implications for the assessment of fraud, including adulterations such as undeclared addition of mammalian or poultry species, which are not amplified by these primer systems. The method provides strictly qualitative data and is not suitable for acquiring quantitative results. For this purpose, multiplex real-time PCR may be employed as previously described [71].

Among the quality criteria outlined by Giusti et al. [72], this study implemented blanks, replicate analysis, genetic database customization, and data filtering. Based on our investigation, the method presented in this study is suitable as a screening tool for enforcement of European and local regulations in Austria.

## Figures and Tables

**Table 2:** Taxa displaying identical barcodes of the 16S rDNA marker used in this study.

Taxonomic Class/Subclass	Species with Identical Barcodes
*Malacostraca*	*Jasus tristani*:*Jasus paulensis*
	*Varuna yui*:*Varuna litterata*
	*Lysmata uncicornis*:*Lysmata arvoredensis*
	*Chionoecetes bairdi*:*Chionoecetes opilio*
	*Portunus sayi*:*Portunus segnis*
	*Charybdis riversandersoni*:*Charybdis miles*
	*Heterocarpus corona*:*Heterocarpus gibbosus*
	*Maja squinado*:*Maja brachydactyla*
	*Polybius holsatus*:*Polybius henslowii*
	*Parapenaeus australiensis*:*Parapenaeus ruberoculatus*
	*Metapenaeus brevicornis*:*Metapenaeus dobsoni*
	*Metanephrops velutinus*:*Metanephrops andamanicus*:*Metanephrops sagamiensis*
	*Parapenaeus sextuberculatus*:*Parapenaeus lanceolatus*:*Parapenaeus kensleyi*:*Parapenaeus indicus*:*Parapenaeus fissuroides*
	*Solenocera melantho*:*Solenocera crassicornis*
	*Heterocarpus woodmasoni*:*Heterocarpus fascirostratus*
	*Heterocarpus parvispina*:*Heterocarpus hayashii*:*Heterocarpus ensifer*
*Coleoidea*	*Sepia recurvirostra*:*Sepia madokai*
	*Octopus mimus*:*Octopus hubbsorum*
	*Octopus minor*:*Octopus variabilis*
	*Sepia pharaonis*:*Sepia ramani*
	*Sepiola rondeleti*:*Sepiola intermedia*
	*Sepiella maindroni*:*Sepiella japonica*
	*Uroteuthis singhalensis*:*Uroteuthis duvaucelii*

**Table 3:** Sequencing results for reference samples. Results are based on one or two sequencing runs (n = 1 or 2, one replicate per run) using the iSeq^®^ 100 and MiSeq^®^ platforms. Identical species were identified in each sample by both Sanger sequencing and NGS. In some cases, a species different from the declaration was detected.

Sample ID	Expected Species	Identified Species	Commercial Name (English)	Total Raw Reads	Total Reads Passing the Pipeline	Reads Assigned Correctly
1	*Aristaeopsis edwardsiana*		Carabineros shrimp	15,547	15,154	15,147
2	*Homarus americanus*		American lobster	48,835	48,211	48,151
3	*Homarus americanus*		American lobster	63,977	60,964	60,876
4	*Litopenaeus vannamei*		Whiteleg shrimp	47,678	47,067	46,988
5	*Litopenaeus vannamei*		Whiteleg shrimp	54,231	53,478	53,440
6	*Litopenaeus vannamei*		Whiteleg shrimp	41,712	41,159	41,089
7	*Litopenaeus vannamei*		Whiteleg shrimp	51,514	50,670	50,630
8	*Litopenaeus vannamei*		Whiteleg shrimp	54,555	53,859	53,811
9	*Litopenaeus vannamei*		Whiteleg shrimp	51,965	51,254	51,180
10	*Litopenaeus vannamei*		Whiteleg shrimp	60,082	59,300	59,243
11	*Macrobrachium rosenbergii*		Giant river prawn	60,082	59,300	59,243
13	*Metapenaeus monoceros*	*Ganjampenaeopsis uncta*	Shrimp	64,968	63,738	62,993
14	*Metapenaeus monoceros*	*Ganjampenaeopsis uncta*	Shrimp	69,722	68,488	67,670
15	*Nephrops norvegicus*		Norway lobster	28,662	26,200	26,034
16	*Panulirus argus*		Caribbean spiny lobster	22,358	21,941	21,838
17	*Pandalus borealis*		Northern prawn	30,632	27,409	27,214
18	*Pandalus borealis*		Northern prawn	67,124	64,459	63,946
19	*Paralithodes camtschaticus*		Red king crab	71,559	68,561	66,508
20	*Paralomis granulosa*		Stone crab	72,018	70,886	70,535
21	*Penaeus monodon*		Giant tiger prawn	40,192	38,446	38,358
22	*Penaeus monodon*		Giant tiger prawn	25,434	25,195	25,058
23	*Penaeus monodon*		Giant tiger prawn	41,889	41,352	41,292
24	*Penaeus notalis*	*Penaeus duorarum*	Shrimp	59,698	59,008	58,929
25	*Pleoticus muelleri*		Argentine red shrimp	48,943	48,338	48,102
26	*Procambarus clarkii*		Red swamp crawfish	107,491	96,537	96,278
27	*Doryteuthis gahi*		Patagonian longfin squid	42,872	42,598	42,473
28	*Dosidicus gigas*		Jumbo flying squid	88,759	86,095	85,906
29	*Illex* *argentinus*	*Euprymna hyllebergi*	Small benthic squid	48,697	46,071	45,292
30	*Loligo* *opalescens*	*Doryteuthis* *opalescens*	Opalescent inshore squid	53,342	52,487	52,339
31	*Nototodarus sloanii*		New Zealand arrow squid	72,036	71,311	71,159
32	*Amphioctopus aegina*		Marbled octopus	25,005	21,545	21,511
32	*Octopus maya*		Mexican four-eyed octopus	64,169	63,576	63,421
33	*Octopus vulgaris*		Common octopus	64,050	54,037	53,451
34	*Octopus vulgaris*		Common octopus	21,756	21,549	21,514
35	*Octopus* *vulgaris*	*Sepia pharaonis*	Pharaoh cuttlefish	36,477	36,146	35,791
36	*Sepiella inermis*		Spineless cuttlefish	44,133	43,773	43,553
37	*Todarodes pacificus*		Japanese flying squid	48,252	47,823	47,728
38	*Uroteuthis chinensis*		Taiwanese squid	27,369	25,801	25,686
39	*Uroteuthis duvaucelii*		Indian Ocean squid	55,824	55,028	54,958
40	*Nephrops norvegicus*		Norway lobster	63,454	62,273	62,216
41	*Procambarus clarkii*		Red swamp crawfish	52,094	48,578	48,475
42	*Litopenaeus vannamei*		Whiteleg shrimp	53,205	52,426	52,332
43	*Penaeus semisulcatus*		Green tiger prawn	40,611	39,201	39,095
44	*Macrobrachium rosenbergii*		Giant river prawn	66,803	63,644	63,536
45	*Pollicipes pollicipes*		Goose neck barnacle	60,266	59,677	59,514
46	*Paralithodes camtschaticus*		Red king crab	75,110	71,749	69,515
47	*Panulirus* *argus*	*Panulirus regius*	Royal spiny lobster	58,954	55,575	55,451
48	*Sepia pharaonis*	*Sepia aculeata*	Common cuttlefish	43,778	43,481	43,407
49	*Pandalus borealis*		Northern prawn	54,690	52,463	52,126
50	*Scylla serrata*		Mud crab	48,582	47,526	47,485
51	*Chionoecetes japonicus*		Red snow crab	22,807	22,409	21,818
52	*Doryteuthis gahi*		Patagonian longfin squid	32,920	32,674	32,215
53	*Illex argentinus*		Argentine shortfin squid	32,202	31,887	31,441
54	*Portunus trituberculatus*		Gazami crab	48,542	47,670	47,291
55	*Procambarus clarkii*		Red swamp crawfish	42,194	39,065	38,914
56	*Penaeus monodon*		Giant tiger prawn	30,930	30,667	30,499
57	*Sepia* spp.	*Sepia officinalis*	European common cuttlefish	37,233	36,968	36,826
58	*Dosidicus gigas*		Jumbo flying squid	35,930	35,736	35,630
59	*Sepiella inermis*		Spineless cuttlefish	70,903	69,531	69,492
60	*Litopenaeus stylirostris*		Blue shrimp	26,578	22,497	22,387
61	*Illex illecebrosus*		Northern shortfin squid	124,657	121,117	120,877
62	*Acetes chinensis*		Shrimp	68,967	64,879	63,905
63	*Cancer pagurus*		Edible crab	133,702	127,196	127,093
64	*Varuna* spp.		Crab	19,124	18,759	18,750
65	*Homarus* *gammarus*	*Homarus* *gammarus*	European lobster, American lobster	63,433	54,462	52,088
		*Homarus* *americanus*				2097

**Table 4:** Sequencing results for DNA extract mixtures. DNA extracts were diluted to 5 ng/µL prior to mixing. Results are based on two sequencing runs (n_1_ = 2, one replicate per run for mixtures with minor components ≥5%; n_2_ = 4, two replicates/run for mixtures with minor components <5%). Samples were sequenced on MiSeq^®^, except for mixtures 24–29. These mixtures were sequenced on both MiSeq^®^ and iSeq^®^ 100, resulting in consistent read assignments. Due to enhanced sequencing depth on iSeq^®^ 100, these results are presented. The double line separates crustacean from cephalopod mixtures.

Number of DNA Extract Mixture	Species	Composition (% *w*/*w*)	Total Number of Raw Reads	Total Number of Reads Passing the Pipeline	Reads Assigned Correctly	Reads Assigned Correctly [%]
1	*Litopenaeus* *vannamei*	98	122,138	120,298	118,561	98.6
	*Penaeus monodon*	1.5			151	0.1
	*Panulirus argus*	0.5			1547	1.3
2	*Litopenaeus* *vannamei*	98	113,774	112,035	111,618	99.6
	*Penaeus monodon*	1.5			147	0.1
	*Homarus gammarus*	0.5			241	0.2
3	*Litopenaeus* *vannamei*	98	130,215	128,309	128,105	99.8
	*Penaeus monodon*	1.5			169	0.1
	*Cancer pagurus*	0.5			12	0.009
4	*Litopenaeus* *vannamei*	17	126,700	118,427	7620	6.4
	*Litopenaeus* *stylirostris*	17			1568	1.3
	*Penaeus monodon*	17			1802	1.5
	*Penaeus semisulcatus*	17			91,888	77.6
	*Penaeus duorarum*	17			11,706	9.9
	*Ganjampenaeopsis uncta*	17			3782	3.2
5	*Litopenaeus* *vannamei*	17	105,130	95,483	1886	2.0
	*Homarus americanus*	17			15,457	16.2
	*Panulirus regius*	17			17,167	18.0
	*Scylla serrata*	17			59,366	62.2
	*Procambarus clarkii*	17			1323	1.4
	*Paralithodes camtschaticus*	17			245	0.3
6	*Procambarus clarkii*	97.0	116,394	110,363	107,597	97.5
	*Litopenaeus* *vannamei*	0.5			258	0.2
	*Litopenaeus* *stylirostris*	0.5			47	0.04
	*Penaeus monodon*	0.5			55	0.1
	*Penaeus semisulcatus*	0.5			1996	1.8
	*Penaeus duorarum*	0.5			292	0.3
	*Ganjampenaeopsis uncta*	0.5			88	0.1
7	*Penaeus monodon*	97.0	91,972	88,023	71,001	80.7
	*Litopenaeus* *vannamei*	0.5			428	0.5
	*Homarus americanus*	0.5			3139	3.6
	*Panulirus regius*	0.5			2998	3.4
	*Scylla serrata*	0.5			9959	11.3
	*Procambarus clarkii*	0.5			396	0.4
	*Paralithodes camtschaticus*	0.5			74	0.1
8	*Panulirus argus*	98	157,230	152,906	152,471	99.7
	*Homarus gammarus*	1.5			344	0.2
	*Cancer pagurus*	0.5			1	0.0
9	*Homarus gammarus*	98	127,103	118,266	114,271	96.6
	*Cancer pagurus*	1.5			15	0.01
	*Panulirus argus*	0.5			1257	1.1
10	*Cancer pagurus*	98	104,264	100,767	48,590	48.2
	*Panulirus argus*	1.5			49,345	49.0
	*Homarus gammarus*	0.5			2661	2.6
11	*Gallus gallus*	85	127,236	119,841	0	0.0
	*Litopeneaus* *vannamei*	5			7737	6.5
	*Homarus gammarus*	5			8448	7.0
	*Scylla serrata*	5			103,424	86.3
12	*Sus scrofa*	85	129,275	122,438	0	0.0
	*Litopeneaus* *vannamei*	5			8129	6.6
	*Homarus gammarus*	5			8535	7.0
	*Scylla serrata*	5			105,519	86.2
13	*Sparus aurata*	85	142,005	134,238	0	0.0
	*Litopeneaus* *vannamei*	5			10,185	7.6
	*Homarus gammarus*	5			10,976	8.2
	*Scylla serrata*	5			112,785	84.0
14	*Zea mays*	85	135,635	127,946	0	0.0
	*Litopeneaus* *vannamei*	5			9150	7.2
	*Homarus gammarus*	5			9583	7.5
	*Scylla serrata*	5			108,942	85.1
15	*Mytilus* spp.	85	144,115	135,886	12	0.009
	*Litopeneaus* *vannamei*	5			9849	7.2
	*Homarus gammarus*	5			9718	7.2
	*Scylla serrata*	5			116,043	85.4
16	*Octopus vulgaris*	98	107,278	105,994	105,783	99.8
	*Illex argentinus*	1.5			70	0.1
	*Uroteuthis chinensis*	0.5			114	0.1
17	*Octopus vulgaris*	98	122,817	121,388	121,204	99.8
	*Illex argentinus*	1.5			82	0.1
	*Dosidicus gigas*	0.5			84	0.1
18	*Octopus vulgaris*	98	121,814	120,142	120,081	99.9
	*Illex argentinus*	1.5			39	0.03
	*Illex illecebrosus*	0.5			0	0.0
19	*Dosidicus gigas*	20	128,539	122,392	64,807	53.0
	*Illex illecebrosus*	20			80	0.1
	*Illex argentinus*	20			12,985	10.6
	*Nototodarus sloanii*	20			11,997	9.8
	*Todarodes pacificus*	20			32,431	26.5
20	*Octopus vulgaris*	20	145,905	142,396	21,957	15.4
	*Dosidicus gigas*	20			17,888	12.6
	*Uroteuthis chinensis*	20			32,115	22.6
	*Euprymna hyllebergi*	20			5783	4.1
	*Sepia pharaonis*	20			63,822	44.8
21	*Illex argentinus*	98.0	97,750	95,347	87,013	91.3
	*Sepia pharaonis*	0.5			5418	5.7
	*Sepia aculeata*	0.5			2642	2.8
	*Sepia officinalis*	0.5			40	0.04
	*Sepiella inermis*	0.5			83	0.1
22	*Illex argentinus*	98.0	79,723	78,069	69,180	88.6
	*Dosidicus gigas*	0.5			1125	1.4
	*Uroteuthis chinensis*	0.5			2165	2.8
	*Euprymna hyllebergi*	0.5			302	0.4
	*Sepia pharaonis*	0.5			4591	5.9
23	*Octopus vulgaris*	98	102,779	101,276	101,229	100.0
	*Illex argentinus*	1.5			43	0.04
	*Uroteuthis duvaucellii*	0.5			1	0.0
24	*Illex argentinus*	98	95,406	94,229	93,283	99.0
	*Uroteuthis duvaucellii*	1.5			83	0.1
	*Octopus vulgaris*	0.5			806	0.9
25	*Uroteuthis duvaucellii*	98	93,731	92,020	58,954	64.1
	*Octopus vulgaris*	1.5			31,270	34.0
	*Illex argentinus*	0.5			1780	1.9
26	*Doryteuthis gahi*	90	131,746	127,458	79,158	62.1
	*Illex argentinus*	5			26,022	20.4
	*Nototodarus sloanii*	5			22,214	17.4
27	*Uroteuthis chinensis*	95	144,194	142,628	142,500	99.9
	*Uroteuthis duvaucellii*	5			55	0.04
28	*Uroteuthis duvaucellii*	95	146,272	142,370	39,983	28.1
	*Uroteuthis chinensis*	5			102,311	71.9
29	*Sepiella inermis*	70	132,448	128,272	22,816	17.8
	*Sepia pharaonis*	10			36,878	28.7
	*Sepia officinalis*	10			559	0.4
	*Sepia aculeata*	10			67,522	52.6
30	*Gallus gallus*	70	154,842	151,040	0	0.0
	*Todarodes pacificus*	5			21,458	14.2
	*Doryteuthis gahi*	5			854	0.6
	*Dosidicus gigas*	5			51,473	34.1
	*Illex argentinus*	5			9402	6.2
	*Octopus vulgaris*	5			66,491	44.0
	*Doryteuthis* *opalescens*	5			2102	1.4
31	*Sus scrofa*	70	108,057	105,584	2211	2.1
	*Todarodes pacificus*	5			15,134	14.3
	*Doryteuthis gahi*	5			625	0.6
	*Dosidicus gigas*	5			35,496	33.6
	*Illex argentinus*	5			5791	5.5
	*Octopus vulgaris*	5			45,054	42.7
	*Doryteuthis* *opalescens*	5			1254	1.2
32	*Gadus* *chalcogrammus*	70	123,571	121,128	112	0.1
	*Todarodes pacificus*	5			18,315	15.1
	*Doryteuthis gahi*	5			805	0.7
	*Dosidicus gigas*	5			42,108	34.8
	*Illex argentinus*	5			6796	5.6
	*Octopus vulgaris*	5			50,836	42.0
	*Doryteuthis* *opalescens*	5			1915	1.6
33	*Zea mays*	70	147,666	144,660	0	0.0
	*Todarodes pacificus*	5			22,409	15.5
	*Doryteuthis gahi*	5			985	0.7
	*Dosidicus gigas*	5			52,820	36.5
	*Illex argentinus*	5			7720	5.3
	*Octopus vulgaris*	5			58,564	40.5
	*Doryteuthis* *opalescens*	5			2136	1.5
34	*Mytilus* spp.	70	136,793	133,560	0	0.0
	*Todarodes pacificus*	5			21,154	15.8
	*Doryteuthis gahi*	5			879	0.7
	*Dosidicus gigas*	5			49,795	37.3
	*Illex argentinus*	5			7209	5.4
	*Octopus vulgaris*	5			52,486	39.3
	*Doryteuthis* *opalescens*	5			1979	1.5

**Table 5:** Samples 1–3 and 4–7 were analyzed in two proficiency tests aimed at detecting food allergens. Sample 3 was a spiked sample and therefore consisted of a different matrix than samples 1 and 2. The displayed results were obtained from a single sequencing run on the MiSeq^®^ platform.

Sample	Food Matrix	Correct Species	Spike Level [mg/kg] ^1^	Identified Species	Total Number of Raw Reads	Reads Passing the Pipeline	Reads Assigned Correctly
1	Spice cracker, baked	*Litopenaeus vannamei*	71.8	*Litopenaeus vannamei*	88,680	84,810	84,802
2	Spice cracker, baked	-	0	-	813	529	522
3	Potato powder	*Litopenaeus vannamei*	30.8	*Litopenaeus vannamei*	262,994	251,942	251,806
4	Potato powder, maltodextrin	-	0	-	99	-	-
5		*Procambarus clarkii*	79	*Procambarus clarkii*	36,462	30,704	30,633
6		*Procambarus clarkii*	151	*Procambarus clarkii*	300,251	250,255	250,189
7		-	0	-	355	88	53

^1^ Amounts (% *w*/*w*) were calculated from spike levels [mg/kg] by converting to g/g (×10^−6^) and then dividing by 100.

**Table 6:** Sequencing results for commercial processed foods containing crustaceans and/or cephalopods. Samples were sequenced on the MiSeq^®^ or iSeq^®^ 100 platform. For products containing both taxa, results for crustaceans are listed first and separated from results for cephalopods by underlining.

Sample ID	Food Product	Species Labeled (Amount in %, if available)	Identified Species	Total Number of Raw Reads	Total Number of Reads Passing the Pipeline	Reads Assigned to the Identified Species
1	Dried shrimps	*Dendrobranchiata*	*Xiphopenaeus kroyeri*	92,985	88,907	85,089
			*Xiphopenaeus riveti*			1638
			*Xiphopenaeus baueri*			1110
2	Squid in squid ink sauce	*Dosidicus gigas*	*Dosidicus gigas*	100,811	100,052	99,915
3	Fried calamari	Calamari	*Doryteuthis gahi*	64,820	64,140	63,931
4	Octopus carpaccio	Octopus	*Octopus cyanea*	74,303	73,567	73,347
5	Squid in squid ink sauce	Squid	*Todarodes pacificus*	111,201	109,956	108,889
6	Seafood mix (bivalves, crustaceans, cephalopods)	*Litopenaeus* *vannamei*, *Uroteuthis* *duvaucelii*, *Mytilus chinensis*	*Litopenaeus vannamei*	83,012 62,214	80,705 60,364	80,683
			*Uroteuthis duvaucelii*			24,760
			*Todarodes pacificus*			20,679
			*Uroteuthis* spp.			7601
			*Uroteuthis edulis*			7170
7	Seafood mix (bivalves, crustaceans, cephalopods)	*Litopenaeus* *vannamei*, *Mytilus chilenis*, *Mytilus edulis*, *Illex argentinus*	*Litopenaeus vannamei*	102,180	100,944	100,651
			*Illex argentinus*	50,803	50,022	49,981
8	Seafood mix (bivalves, crustaceans, cephalopods)	*Dosidicus gigas*, *Octopus* *membranaceus*, *Mytilus* *galloprovincialis*, *Litopenaeus* *vannamei*	*Dosidicus gigas*	90,584	85,748	85,731
9	Seafood mix (bivalves, crustaceans, cephalopods)	*Mytilus chilensis*, *Paphia undulata*, *Penaeus vannamei*, *Loligo duvaucelii*	*Litopenaeus vannamei*	104,482	102,594	101,889
			*Uroteuthis duvaucelii*			35,769
			*Uroteuthis* spp.	63,647	61,670	22,312
			*Uroteuthis edulis*			3543
10	Frozen shrimp	*Penaeus* *merguiensis*, *Metapenaeus ensis*, *Litopenaeus* *vannamei*	*Fenneropenaeus* *merguiensis*	86,435	85,727	85,035
11	Soup cube (prawn soup)	Norway lobster (*Nephrops* *norvegicus*)	-	65,778	328	-
12	Prawn cracker	*Penaeus* *merguiensis*	*Litopenaeus vannamei*	98,952	98,145	93,297
			*Euphausia superba*			4473
13	Pesto from Styrian mountain prawn and basil	*Litopenaeus* *stylirostris* (22%)	*Litopenaeus vannamei*	105,687	104,996	104,884
14	Nero di Sepia, squid ink	*Sepia officinalis*	*Sepia officinalis*	80,296	79,274	72,599
			*Sepia* spp.			3603
			*Sepia pharaonis*			2050
			*Sepia hierredda*			812
15	Seafood mix (bivalves, crustaceans, cephalopods)	*Litopenaeus vannamei* (1) or Argentine red shrimp *Pleoticus muelleri* (2) (A)/ *Uroteuthis duvaucelii* (1), *Dosidicus gigas* (2), *Sepia pharaonis* (3), *Sepia aculeata* (4) *Illex argentinus* (5), *Nototodarus sloanii* (6) (B)/*Mytilus* *chilensis*	*Litopenaeus vannamei*	94,825 61,573	93,555 54,620	92,040
			*Ganjampenaeopsis uncta*			1003
			*Sepia* spp.			20,170
			*Sepia pharaonis*			13,875
			*Uroteuthis duvaucelii*			9975
			*Todarodes pacificus*			6141
			*Uroteuthis edulis*			3786
			*Uroteuthis* spp.			519
			*Illex argentinus*			134
16	Squid rings	*Illex argentinus*	*Illex argentinus*	46,295	45,697	45,520
17	Dried red shrimp	*Dendrobranchiata*, shrimp	*Litopenaeus vannamei*	132,346	130,570	130,367
18	Ground crayfish	Crayfish	*Nematopalaemon schmitti*	305,241	272,628	268,128
			*Lysmata* spp.			3988
19	Linguine with squid ink	Sepia (0.86%)	*Sepia officinalis*	45,180	43,341	36,830
			*Sepia hierredda*			6387
20	Spaghetti with squid ink	Sepia	*Sepia ramani*	51,791	50,253	31,561
			*Sepia* spp.			9775
			*Sitophilus oryzae*			5852
			*Sepia hierredda*			2951
21	Lobster butter	*Homarus* *americanus* (24%)	*Homarus americanus*	80,294	78,614	78,362
22	Crab butter	*Cancer pagurus* (25%)	*Cancer pagurus*	90,220	89,093	88,854
23	Prawn butter	*Pandalus borealis* (28%)	*Pandalus borealis*	94,127	88,767	88,003
24	Crab creme	*Cancer pagurus* (23%)	*Cancer pagurus*	69,045	68,241	67,971
25	Squid in olive oil	*Dosidicus gigas* (65%)	*Dosidicus gigas*	92,687	91,984	91,904
26	Filled squid in olive oil	*Loligo* spp.	*Doryteuthis pealei*	84,890	83,381	71,779
			*Uroteuthis duvaucelii*			11,471
27	Instant noodle soup, shrimp flavor	Shrimp (0.2%)	*Acetes chinensis*	71,563	69,380	66,777
			*Acetes japonicus*			1384
28	Lobster soup (lobster, Norway lobster, shrimp powder)	*Homarus* *americanus* (2.2%), *Nephrops* *norvegicus* (1.9%), shrimp powder	*Homarus* *americanus*	95,534	93,587	90,792
			*Nephrops norvegicus*			2544
29	Instant noodle soup (spicy seafood flavor) *	Shrimp (0.15%)	*Litopenaeus* *vannamei*	73,660	72,383	55,860
			*Acetes japonicus*			10,633
			*Macrobrachium* *lanchesteri*			2722
			*Mesopodopsis* *orientalis*			936
			*Acetes indicus*			871
			*Acetes* spp.			541
			*Procletes levicarina* ^a^			352
30			*Acetes chinensis*	65,860	63,603	60,193
			*Oratosquillina* *perpensa*			1136
			*Acetes japonicus*			926
			*Oratosquilla oratoria*			713
			*Oratosquillina* *interrupta* ^b^			436
31	Octopus in chimichurri sauce	Octopus	*Octopus vulgaris*	79,255	78,415	78,223
32	Sugo Pronto al Nero di Sepia	Calamari, squid ink (1.2%)	*Dosidicus gigas*	21,026	2261	2145
33	Seafood stew	Calamari, Musk octopus, Sepia	*Sepia* spp.	132,776	131,143	126,017
			*Amphioctopus aegina*			4866
34	Fried gambas in garlic oil	Gambas	*Pleoticus muelleri*	71,984	71,393	71,264
35	Prawn crackers	Shrimp (10%)	*Acetes indicus*	121,032	119,917	66,184
			*Litopenaeus vannamei*			40,284
			*Acetes* spp.			8665
			*Acetes japonicus*			4652
36	Shrimp chips	Shrimp (20%)	*Ganjampenaeopsis uncta*	123,668	121,526	35,492
			*Metapenaeus affinis*			32,642
			*Metapenaeus ensis*			27,061
			*Penaeidae*			16,429
			*Alcockpenaeopsis* *hungerfordii*			5156
			*Fenneropenaeus* *merguiensis*			3572
			*Fenneropenaeus* *penicillatus*			816
37	Canned spider crab meat	*Maja squinado*	*Maja* spp.	87,205	85,694	83,890
			*Maja crispata*			1665
38	Canned snow crab meat	*Chionoecetes apilio*	*Chionoecetes* spp.	55,109	54,561	54,101
39	Breaded squid rings	Squid	*Dosidicus gigas*	85,936	85,240	85,142
40	Frozen king prawns	*Litopenaeus* *vannamei*	*Litopenaeus vannamei*	53,500	50,677	50,667
41	Frozen shrimps	Shrimps	*Litopenaeus vannamei*	53,188	49,818	49,765
42	Frozen shrimps	*Litopenaeus* *vannamei*	*Litopenaeus vannamei*	59,840	56,703	56,658
43	Frozen black tiger shrimp	*Penaeus monodon*	*Penaeus monodon*	48,142	44,297	44,185
44	Frozen shrimps	Shrimps	*Litopenaeus vannamei*	53,843	51,196	51,183
45	Cooked octopus	*Octopus vulgaris*	*Octopus vulgaris*	42,871	41,587	41,387
46	Cooked crayfish tails with dill, frozen	*Procambarus clarkii*	*Procambarus clarkii*	60,948	52,506	52,232
47	Frozen giant squid tentacles	*Dosidicus gigas*	*Dosidicus gigas*	52,098	51,026	51,008
48	Frozen king prawns	*Litopenaeus* *vannamei*	*Litopenaeus vannamei*	53,936	51,322	51,316
49	Frozen shrimp	*Litopenaeus* *vannamei*	*Litopenaeus vannamei*	59,845	56,949	56,920
50	Frozen king prawns	*Litopenaeus* *vannamei*	*Litopenaeus vannamei*	55,553	52,670	52,619
51	Frozen white tiger shrimps	*Litopenaeus* *vannamei*	*Litopenaeus vannamei*	66,902	63,700	63,688
52	White tiger shrimps	*Litopenaeus* *vannamei*	*Litopenaeus vannamei*	55,412	52,101	52,084
53	Frozen black tiger shrimp	*Penaeus monodon*	*Litopenaeus vannamei*	30,879	28,760	28,203
54	Cooked squid tentacle slices, frozen	*Dosidicus gigas*	*Dosidicus gigas*	47,944	47,160	47,115
55	Frozen shrimp	*Litopenaeus* *vannamei*	*Litopenaeus vannamei*	50,563	48,711	48,699
56	Frozen shrimp	*Litopenaeus* *vannamei*	*Litopenaeus vannamei*	55,110	52,296	52,243
57	Frozen black tiger shrimp	*Penaeus monodon*	*Penaeus monodon*	54,049	51,034	50,859
58	Frozen shrimp	*Litopenaeus* *vannamei*	*Litopenaeus vannamei*	62,333	59,871	59,827
59	Frozen shrimp	*Litopenaeus* *vannamei*	*Litopenaeus vannamei*	53,424	51,387	51,368
60	Frozen shrimp	*Litopenaeus* *vannamei*	*Litopenaeus vannamei*	62,691	60,273	60,266
61	Frozen shrimp	*Litopenaeus* *vannamei*	*Litopenaeus vannamei*	52,674	50,362	50,256
62	Frozen shrimp	*Litopenaeus* *vannamei*	*Litopenaeus vannamei*	48,023	46,249	46,213
63	Frozen shrimp	*Litopenaeus* *vannamei*	*Litopenaeus vannamei*	49,720	47,929	49,887
64	Frozen shrimp	*Litopenaeus* *vannamei*	*Litopenaeus vannamei*	52,318	49,921	49,887
65	Frozen shrimp	*Litopenaeus* *vannamei*	*Litopenaeus vannamei*	53,908	50,124	50,083
66	Frozen squid	*Uroteuthis* *duvaucelii*	*Uroteuthis duvaucelii*	49,152	48,187	47,703
			*Uroteuthis edulis*			286
67	Frozen shrimp	*Crangon* *crangon*	*Carcinus maenas*	22,143	21,684	10,113
			*Palaemon* *macrodactylus*			5887
			*Palaemon serratus*			5479
68	Frozen shrimp	*Litopenaeus* *vannamei*	*Fenneropenaeus* *merguiensis*	48,844	48,022	35,530
			*Fenneropenaeus* *penicillatus*			12,367
69	Canned swimming crab meat	*Portunus* spp.	*Monomia gladiator*	55,299	53,149	22,645
			*Portunus* *sanguinolentus*			18,062
			*Monomia lucida*			5596
			*Charybdis natator*			3284
			*Portunus* *gracilimanus*			2240
70	Canned swimming crab meat	*Portunus* spp.	*Ovalipes punctatus*	51,621	46,285	35,434
			*Charybdis natator*			5077
			*Monomia gladiator*			3161
			*Portunus* *sanguinolentus*			2189
71	Frozen shrimp	Shrimps	*Metapenaeopsis* *palmensis*	33,992	29,241	18,626
			*Alcockpenaeopsis*			4924
			*hungerfordii*			
			*Parapenaeopsis* *hardwickii*			2761
			*Solenocera* spp.			2676
72	Frozen squid	*Uroteuthis* *duvaucelii*	*Uroteuthis duvaucelii*	35,720	35,181	29,156
			*Uroteuthis* spp.			5896
73	Frozen squid	*Loligo edulis*	*Uroteuthis chinensis*	55,942	49,906	28,721
			*Uroteuthis duvaucelii*			16,239
			*Uroteuthis edulis*			4858
74	Frozen squid	*Uroteuthis edulis*	*Octopus cyanea*	31,410	28,266	12,849
			*Euprymna hyllebergi*			6143
			*Amphioctopus* *marginatus*			4439
			*Amphioctopus aegina*			4310
75	Cooked shrimp	*Crangon crangon*	*Litopenaeus vannamei*	68,223	66,313	55,877
			*Pandalus borealis*			8939
76	Blanched, frozen shrimp	*Metapenaeus* *affinis*	*Metapenaeus* *monoceros*	48,500	47,730	44,337
			*Metapenaeus affinis*			2768
77	Peeled shrimp	*Crangon* *crangon*	*Loligo vulgaris*	116,997	111,662	65,977
			*Liocarcinus* *marmoreus*			23,297
			*Liocarcinus holsatus*			22,176
78	Peeled shrimp	*Crangon* *crangon*	*Liocarcinus holsatus*	122,767	112,657	58,184
			*Liocarcinus* *marmoreus*			34,381
			*Metapenaeus ensis*			19,663
79	Frozen squid	*Loligo edulis*	*Uroteuthis chinensis*	104,561	78,626	50,760
			*Uroteuthis duvaucelii*			27,780
80	Frozen squid	*Uroteuthis* *duvaucelii*	*Uroteuthis edulis*	102,839	97,739	89,516
			*Uroteuthis duvaucelii*			8143
81	Frozen squid	*Uroteuthis edulis*	*Uroteuthis chinensis*	80,388	64,314	55,573
			*Uroteuthis duvaucelii*			8667
82	Cooked shrimp	*Crangon* *crangon*	*Pandalus montagui*	80,414	78,400	78,295
83	Cooked shrimp	*Crangon* *crangon*	*Pandalus montagui*	31,892	19,995	19,772
84	Cooked shrimp	*Heterocarpus redii*	*Heterocarpus sp. S6-2*	60,640	57,394	57,302
85	Fresh shrimp	*Macrobrachium* *rosenbergii*	*Penaeus monodon*	55,191	54,328	54,065
86	Fresh shrimp	*Penaeus* *occidentalis*	*Pleoticus muelleri*	61,182	60,371	60,261
87	Squid in oil	*Eledone moschata*	*Amphioctopus aegina*	67,626	67,021	66,561
88	Frozen squid	*Loligo edulis*	*Uroteuthis chinensis*	54,972	54,437	54,253
89	Frozen sepia	*Sepiella japonica*	*Sepiella inermis*	71,160	70,577	70,373
90	Fresh squid	*Sepia officinalis*	*Sepia pharaonis*	63,447	62,979	62,877
91	Frozen squid	*Loligo chinensis*	*Doryteuthis gahi*	53,813	53,368	53,279
92	Fresh shrimp	*Litopenaeus* *stylirostris*	*Litopenaeus vannamei*	56,813	56,033	55,947
93	Fresh squid	Calamari	*Doryteuthis gahi*	60,008	59,541	59,412
94	Frozen crab	*Portunus* *pelagicus*	*Portunus* *trituberculatus*	50,738	46,460	46,072
95	Frozen baby shrimp	*Metapenaeus* spp.	*Acetes chinensis*	33,897	33,599	33,079
96	Frozen shrimp	*Penaeus monodon*	*Litopenaeus vannamei*	32,468	30,509	30,434
97	Frozen crab	*Thenus orientalis*	*Thenus unimaculatus*	38,568	36,506	35,999
98	Frozen softshell crab	*Scylla serrata*	*Scylla olivacea*	26,349	24,163	24,077
99	Frozen crayfish	Crayfish	*Astacus leptodactylus*	33,698	28,395	28,242
100	Frozen squid	*Loligo edulis*	*Uroteuthis chinensis*	110,023	107,066	106,953
101	Cooked, frozen shrimp	*Heterocarpus reedi*	*Heterocarpus sp. S6-2*	51,962	46,474	46,224
102	Frozen crab	*Scylla serrata*	*Scylla olivacea*	39,524	30,476	30,267
103	Frozen shrimp	*Macrobrachium* *rosenbergii*	*Penaeus monodon*	46,195	45,246	45,245
104	Frozen crayfish	Crayfish	*Procambarus clarkii*	74,346	69,883	69,883
105	Frozen squid rings	Squid	*Illex argentinus*	55,705	54,753	54,730
106	Fresh giant prawn	*Aristaeomorpha* *foliacea*	*Aristaeopsis* spp.	35,422	29,967	26,964

* Sample included two separate components, seasoning ^a^ and flavor oil ^b^, each extracted and analyzed separately.

## Data Availability

The datasets generated during the current study are available from the corresponding authors on reasonable request.

## Supplementary Materials

The following supporting information can be downloaded at https://www.mdpi.com/article/10.3390/foods14091549/s1, Figure S1. Comparison of predicted hairpin structures in single-stranded DNA for *Dosidicus gigas* (a,b) and *Illex illecebrosus* (c,d). Forward primer binding sites are indicated in panels (a,c); reverse primer binding sites in panels (b,d). While the overall structures are similar, *D. gigas* exhibits less favorable configurations, including longer hairpin stems [62]. Nevertheless, *I. illecebrosus* was either not or minimally detected in DNA extract mixtures containing species other than *D. gigas*; Table S1: List of squid and crustacean mitochondrial 16S rDNA sequences obtained from the NCBI database for inclusion in the AGES customized database (including accession numbers and scientific species names). Accession numbers in the format “WS_0000XY.1” represent sequences generated in this study and incorporated into the customized database following species confirmation via BLASTn analysis. This appendix was compiled in January 2025; NCBI accession numbers may be subject to change over time.
