# Supplementary material for: Development of a DNA Metabarcoding Method for the Identification of Crustaceans (Malacostraca) and Cephalopods (Coleoidea) in Processed Foods
Source: Foods. 2025 Apr 28;14(9):1549. doi: 10.3390/foods14091549 (PMC12071261; doi:10.3390/foods14091549)
Supplement: Supplementary file 1 [file foods-14-01549-s001.zip › foods-3568483-Supplementary.pdf]

Appendix A

Supplementary Table S1: List of squid and crustacean mitochondrial 16S rDNA sequences obtained from the NCBI database for inclusion in the AGES customized database (including accession numbers and scientific species names). Accession numbers in the format "WS_0000XY.1" represent sequences generated in this study and incorporated into the customized database following species confirmation via BLASTn analysis. This appendix was compiled in January 2025; NCBI accession numbers may be subject to change over time.

Accession Number	Scientific name (Latin)
AB_191138.1	Rossia pacifica
AB_197677.1	Erimacrus isenbeckii
AB_270948.1	Illex illecebrosus
AB_270953.1	Nototodarus sloanii
AB_451090.1	Mesopodopsis orientalis
AF_107608.1	Alachosquilla vicina
AF_110075.2	Loligo forbesi
AF_110076.2	Doryteuthis gahi
AF_110082.2	Loligo vulgaris
AF_175234.1	Parastacus nicoleti
AF_175243.1	Parastacus defossus
AF_175247.1	Parastacus pilimanus
AF_192869.1	Jasus caveorum
AF_192872.1	Jasus spp. (same barcode for AF_192872.1 Jasus tristani and AF_192870.1 Jasus paulensis)
AF_279822.1	Melicertus plebejus
AF_425333.1	Lopholithodes mandtii
AF_502949.1	Scyllarus arctus
AF_502955.1	Palinurus elephas
AF_502956.1	Jasus edwardsii
AJ_252763.1	Eledone massyae
AJ_252764.1	Eledone moschata
AJ_252765.1	Eledone palari
AJ_298180.1	Callinectes bocourti
AJ_784020.2	Episesarma mederi
AM_088005.1	Sepia elliptica
AY_151823.1	Eriocheir recta
AY_293666.1	Sepiola birostrata
AY_293671.1	Sepiola spp. (same barcode for AY_293671.1 Sepiola rondeleti and MW_478839.1 Sepiola intermedia)
AY_293672.1	Sepiola atlantica
AY_293676.1	Rossia bipillata
AY_351244.1	Cervimunida johni
AY_368675.1	Sepia hierredda
AY_368678.1	Sepia bertheloti
AY_514042.1	Pollicipes mitella
AY_545113.1	Sepiola sp. JMS-2004

AY_583901.1	Carcinus maenas
AY_595927.1	Lithodes santolla
AY_612870.1	Heterocarpus amacula
AY_612878.1	Heterocarpus intermedius
AY_616973.1	Eledone cirrhosa
AY_686590.1	Loligo pealei
AY_947836.1	Pseudosquilla ciliata
CM_029732.1	Pollicipes pollicipes
DQ_062208.1	Palinurus mauritanicus
DQ_079709.1	Carcinus maenas
DQ_079723.1	Maja spp. (same barcode for DQ_079723.1 Maja squinado and MN_006157.1 Maja brachydactyla)
DQ_079730.1	Panulirus regius
DQ_194960.1	Macrobrachium yui
DQ_377975.1	Palinurus charlestoni
DQ_388058.1	Portunus floridanus
DQ_388061.1	Portunus spinicarpus
DQ_407679.1	Callinectes ornatus
DQ_407680.1	Callinectes danae
DQ_681290.1	Macrobrachium australe
EF_060259.1	Paranephrops zealandicus
EF_490009.1	Macrobrachium faustinum
EF_546316.1	Palinurus delagoae
EF_546321.1	Palinurus gilchristi
EF_546339.1	Palinurus barbarae
EF_588317.1	Macrobrachium australiense
EF_588321.1	Macrobrachium olfersii
EF_599137.1	Parastacus pugnax
EU_186109.1	Metanephrops binghami
EU_186112.1	Metanephrops spp. (same barcode for EU_186112.1 Metanephrops velutinus, EU_186113.1 Metanephrops andamanicus and EU_186111.1 Metanephrops sagamiensis)
EU_186115.1	Metanephrops japonicus
EU_186116.1	Metanephrops armatus
EU_186117.1	Metanephrops formosanus
EU_186118.1	Metanephrops sinensis
EU_186121.1	Metanephrops boschmai
EU_186123.1	Metanephrops arafurensis
EU_186127.1	Metanephrops neptunus
EU_186128.1	Metanephrops challengeri
EU_221186.1	Jasus lalandii
EU_493137.1	Macrobrachium asperulum
EU_493149.1	Macrobrachium equidens
EU_735192.1	Rossia palpebrosa
EU_735218.1	Illex coindetii
EU_735263.1	Berryteuthis magister

EU_735265.1	Gonatopsis okutanii
EU_882877.1	Metanephrops rubellus
EU_920933.1	Parastacus varicosus
FJ_152159.1	Portunus hastatus
FJ_174906.1	Scyllarides herklotsii
FJ_174908.1	Scyllarus pygmaeus
FJ_174909.1	Scyllarus caparti
FJ_224251.1	Anchisquilla fasciata
FJ_224254.1	Clorida decorata
FJ_224270.1	Miyakea nepa
FJ_224281.1	Oratosquillina interrupta
FJ_224282.1	Odontodactylus japonicus
FJ_435639.1	Metapenaeus moyebi
FJ_459771.1	Stomatopoda sp. 2 RWKT-2009
FJ_871138.1	Kempina mikado
FJ_965952.1	Astacoides madagascariensis
FM_208751.1	Portunus ordwayi
FM_208752.1	Portunus inaequalis
GQ_302759.1	Heterocarpus lepidus
GQ_302761.1	Heterocarpus laevigatus
GQ_412305.1	Nototodarus gouldi
HM_015408.1	Thenus parindicus
HM_015412.1	Thenus australiensis
HM_020978.1	Paralomis granulosa
HM_138813.1	Alima orientalis
HM_138831.1	Haptosquilla trispinosa
HM_138847.1	Pullosquilla thomassini
HM_352437.1	Macrobrachium jelskii
HM_352441.1	Macrobrachium amazonicum
HM_352447.1	Macrobrachium americanum
HM_637974.1	Menippe mercenaria
HQ_845987.1	Abralia andamanica
JN_566195.1	Jasus frontalis
JN_701692.1	Scyllarides nodifer
JN_701698.1	Ibacus chacei
JN_701700.1	Ibacus peronii
JN_701732.1	Scyllarus americanus
JN_701734.1	Scyllarus chacei
JN_701735.1	Scyllarus depressus
JQ_229876.1	Puerulus sewelli
JQ_229878.1	Thenus indicus
JQ_229901.1	Thenus unimaculatus
JQ_390474.1	Macrobrachium dienbienphuense
JQ_805806.1	Macrobrachium digueti
KC_507002.1	Macrobrachium lar
KC_951896.1	Uroteuthis edulis

GQ_302727.1	Heterocarpus spp. (same barcode for GQ_302727.1 Heterocarpus corona and GQ_302747.1 Heterocarpus gibbosus)
KF_220511.1	Charybdis lucifera
KF_636899.1	Macrobrachium occidentale
KF_854044.1	Uroteuthis duvauceli
KF_953965.1	Melicertus kerathurus
KJ_605236.1	Octopus tetricus
KM_074036.1	Haptosquilla hamifera
KP_059267.1	Parapenaeus americanus
KP_059269.1	Parapenaeus spp. (same barcode for KP_059269.1 Parapenaeus australiensis and KP_059268.1 Parapenaeus ruberoculatus)
KP_059270.1	Parapenaeus cayrei
KP_059273.1	Parapenaeus fissurus
KP_059274.1	Parapenaeus investigatoris
KP_059278.1	Parapenaeus longipes
KP_059280.1	Parapenaeus murrayi
KP_059281.1	Parapenaeus perezfarfantae
KP_059282.1	Parapenaeus politus
KP_059283.1	Parapenaeus spp. (same barcode for KP_059283.1 Parapenaeus sextuberculatus, KP_059276.1 Parapenaeus lanceolatus, KP_059275.1 Parapenaeus kensleyi, KP_059272.1 Parapenaeus indicus and KP_059271.1 Parapenaeus fissuroides)
KP_059284.1	Parapenaeopsis cornuta
KP_178984.1	Palaemon macrodactylus
KP_725528.1	Heterocarpus dorsalis
KP_725530.1	Heterocarpus sibogae
KP_725531.1	Heterocarpus spp. (same barcode for KP_725531.1 Heterocarpus woodmasoni and MK_470780.1 Heterocarpus fascirostratus)
KP_763702.1	Macrobrachium idella
KR_026905.1	Portunus pseudohastatoides
KR_084324.1	Metanephrops mozambicus
KR_736341.1	Macrobrachium malcolmsonii
KT_365554.1	Portunus arabicus
KT_365556.1	Portunus longispinosus
KT_365559.1	Portunus ventralis
KT_365594.1	Charybdis acuta
KT_365604.1	Portunus anceps
KT_365605.1	Portunus granulatus
KT_365606.1	Portunus petreus
KT_365607.1	Portunus spp. (same barcode for KT_365607.1 Portunus sayi and KU_296938.1 Portunus segnis)
KT_372710.1	Heterocarpus chani
KT_959508.1	Penaeus setiferus
KU_532327.1	Erugosquilla woodmasoni
KU_560482.1	Liocarcinus marmoreus
KU_560486.1	Polybius spp. (same barcode for KU_560486.1 Polybius holsatus and FJ_152157.1 Polybius henslowii)

KU_985122.1	Pleuroncodes planipes
KX_060401.1	Charybdis spp. (same barcode for KX_060401.1 Charybdis riversandersoni and KX_060394.1 Charybdis miles)
KX_060420.1	Charybdis orientalis
KX_162743.1	Trachysalambria curvirostris
KX_279349.1	Astacus leptodactylus
KX_984332.1	Euprymna hyllebergi
KY_192526.1	Parastacus tuerkayi
KY_236044.1	Manningia pilaensis
KY_236045.1	Bathysquilla crassispinosa
KY_236046.1	Faughnia profunda
KY_236047.1	Faughnia formosae
KY_426332.1	Lithodes aequispinus
KY_426333.1	Lithodes maja
KY_449062.1	Penaeus paulensis
KY_449068.1	Xiphopenaeus kroyeri
KY_449071.1	Xiphopenaeus riveti
KY_449074.1	Xiphopenaeus dincao
KY_449076.1	Xiphopenaeus baueri
KY_492468.1	Pollicipes elegans
LC_121063.1	Sepia spp. (same barcode for KY_978645.1 Sepia recurvirostra and LC_121063.1 Sepia madokai)
LC_150203.1	Metapenaeus monoceros
LC_310712.1	Metapenaeus stebbingi
LC_341266.1	Pandalus borealis
LC_569858.1	Macrobrachium grandimanus
LC_635856.1	Penaeus japonicus
MF_040872.2	Octopus insularis
MF_346762.1	Madeirasquilla tuerkayi
MF_346763.1	Platysquilla eusebia
MF_460396.1	Scyllarus subarctus
MF_490143.1	Penaeus subtilis
MF_490148.1	Scyllarides deceptor
MF_490225.1	Nematopalaemon schmitti
MG_001053.1	Penaeus brasiliensis
MG_029425.1	Heterocarpus calmani
MG_551495.1	Parastacus brasiliensis
MG_563791.1	Monomia lucida
MG_821354.1	Penaeus semisulcatus
MG_999649.1	Enteroctopus megalocyathuy
MG_999655.1	Octopus maya
MH_168230.1	Miyakea holoschista
MH_168232.1	Faughnia serenei
MH_168234.1	Cloridopsis scorpio
MH_168235.1	Lenisquilla lata
MH_168238.1	Oratosquillina asiatica

MH_253070.1	Macrobrachium hobbsi
MH_300640.1	Heterocarpus sp. S6
MK_000270.1	Pandalus jordani
MK_000271.1	Heterocarpus sp. S6-2
MK_000284.1	Crangon crangon
MK_164420.1	Parapenaeopsis tenella
MK_245778.1	Acetes indicus
MK_430861.1	Ganjampenaeopsis uncta
MK_470794.1	Pandalus montagui
MK_470795.1	Pandalus platyceros
MK_500702.1	Metapenaeus spp. (same barcode for MK_500702.1 Metapenaeus brevicornis and MK_500704.1 Metapenaeus dobsoni)
MK_991817.1	Ommastrephes brevimanus
MT_002801.1	Lysmata spp. (same barcode for MT_002801.1 Lysmata uncicornis and MH_142086.1 Lysmata arvoredensis)
MT_131241.1	Metapenaeus lysianassa
MT_155937.1	Alcockpenaeopsis hungerfordii
MT_155958.1	Metapenaeopsis palmensis
MT_192551.1	Charybdis natator
MT_862440.1	Astacus astacus
MW_446891.1	Charybdis granulata
MW_478836.1	Sepiola affinis
MW_478843.1	Sepiola robusta
MW_732701.1	Ommastrephes caroli
MW_864094.1	Dictyosquilla foveolata
MZ_594996.1	Gonodactylaceus falcatus
NC_002184.1	Penaeus monodon
NC_002507.1	Loligo bleekeri
NC_004251.1	Panulirus japonicus
NC_005037.1	Portunus trituberculatus
NC_005936.1	Pollicipes polymerus
NC_006081.1	Squilla mantis
NC_006281.1	Callinectes sapidus
NC_006353.1	Octopus vulgaris
NC_006354.1	Todarodes pacificus
NC_006880.1	Macrobrachium rosenbergii
NC_006916.1	Harpiosquilla harpax
NC_006992.1	Eriocheir sinensis
NC_007442.1	Gonodactylus chiragra
NC_007443.1	Lysiosquillina maculata
NC_007444.1	Squilla empusa
NC_007893.1	Watasenia scintillans
NC_007894.1	Sepioteuthis lessoniana
NC_007895.1	Sepia officinalis
NC_007896.1	Amphioctopus fangsiao
NC_009626.1	Litopenaeus vannamei

NC_009679.1	Penaeus chinensis
NC_009690.1	Sepia esculenta
NC_009734.1	Dosidicus gigas
NC_010636.1	Sthenoteuthis oualaniensis
NC_011243.1	Cherax destructor
NC_011581.1	Architeuthis dux
NC_011597.1	Eriocheir japonica
NC_011598.1	Eriocheir hepuensis
NC_012060.1	Litopenaeus stylirostris
NC_012217.1	Macrobrachium lanchesteri
NC_012565.1	Scylla serrata
NC_012567.1	Scylla tranquebarica
NC_012569.1	Scylla olivacea
NC_012572.1	Scylla paramamosain
NC_012738.1	Farfantepenaeus californiensis
NC_012840.1	Doryteuthis opalescens
NC_013246.1	Charybdis japonica
NC_014339.1	Panulirus stimpsoni
NC_014342.1	Oratosquilla oratoria
NC_014854.1	Panulirus ornatus
NC_015073.1	Macrobrachium nipponense
NC_015607.1	Homarus americanus
NC_015896.1	Octopus spp. (same barcode for NC_015896.1 Octopus minor and NC_038213.1 Ocotpus variabilis)
NC_016015.1	Panulirus homarus
NC_016423.1	Bathyteuthis abyssicola
NC_016425.1	Semirossia patagonica
NC_016926.1	Procambarus clarkii
NC_017600.1	Acetes chinensis
NC_017746.1	Uroteuthis edulis
NC_020020.1	Homarus gammarus
NC_020021.1	Procambarus fallax
NC_020022.1	Scyllarides latus
NC_020029.1	Paralithodes camtschaticus
NC_020348.1	Ommastrephes bartramii
NC_021146.1	Sepia pharaonis
NC_021458.1	Paralithodes brevipes
NC_022466.1	Sepia apama
NC_022467.1	Sepia latimanus
NC_022468.1	Sepia lycidas
NC_022693.1	Sepiella inermis
NC_022936.1	Cherax cainii
NC_022937.1	Cherax quadricarinatus
NC_022938.1	Cherax monticola
NC_022939.1	Cherax glaber
NC_022959.1	Sepia aculeata

NC_023257.1	Cistopus taiwanicus
NC_023478.1	Cherax robustus
NC_023479.1	Cherax quinquecarinatus
NC_023480.1	Cherax dispar
NC_023481.1	Cherax cairnsensis
NC_023482.1	Cherax preissii
NC_024029.1	Cherax crassimanus
NC_024202.1	Lithodes nintokuae
NC_024440.1	Thenus orientalis
NC_024632.1	Charybdis feriata
NC_025323.1	Metanephrops sibogae
NC_025581.1	Ibacus ciliatus
NC_025957.1	Paranephrops planifrons
NC_025958.1	Nephrops norvegicus
NC_026209.1	Portunus pelagicus
NC_026215.1	Astacopsis gouldi
NC_026224.1	Cherax holthuisi
NC_026226.1	Cherax bicarinatus
NC_026227.1	Cherax boesemani
NC_026559.1	Cherax tenuimanus
NC_026560.1	Austropotamobius pallipes
NC_026561.1	Orconectes limosus
NC_026724.1	Loliolus uyii
NC_026834.1	Metapenaeus ensis
NC_026884.1	Fenneropenaeus merguiensis
NC_026885.1	Fenneropenaeus penicillatus
NC_026908.1	Illex argentinus
NC_027178.1	Squilloides leptosquilla
NC_027601.1	Palaemon serenus
NC_027602.1	Macrobrachium bullatum
NC_027608.1	Metanephrops thomsoni
NC_027729.1	Uroteuthis duvaucelii
NC_028024.1	Panulirus cygnus
NC_028034.1	Loliolus beka
NC_028189.1	Uroteuthis chinensis
NC_028225.1	Portunus sanguinolentus
NC_028447.1	Procambarus alleni
NC_028547.1	Octopus bimaculatus
NC_028627.1	Panulirus versicolor
NC_028731.1	Sepiella spp. (same barcode for NC_028731.1 Sepiella maindroni and NC_017749.1 Sepiella japonica)
NC_029240.1	Palaemon gravieri
NC_029702.1	Amphioctopus aegina
NC_029720.1	Orconectes rusticus
NC_029721.1	Orconectes sanbornii
NC_029723.1	Octopus bimaculoides

NC_029747.1	Octopodoidea (same barcode for NC_029747.1 Octopus conispadiceus and NC_056385.1 Enteroctopus dofleini)
NC_030208.1	Loliolus japonica
NC_030255.1	Munida gregaria
NC_030277.1	Parapenaeopsis hardwickii
NC_030765.1	Sitophilus oryzae
NC_030768.1	Orconectes punctimanus
NC_031366.1	Penaeus indicus
NC_033504.1	Austropotamobius torrentium
NC_033508.1	Orconectes luteus
NC_033509.1	Pacifastacus leniusculus
NC_033510.1	Procambarus acutus
NC_035424.1	Maja crispata
NC_036351.1	Amphioctopus marginatus
NC_037155.1	Varuna spp. (same barcode for NC_037155.1 Varuna yui and NC_056882.1 Varuna litterata)
NC_037173.1	Monomia gladiator
NC_037695.1	Charybdis bimaculata
NC_038069.1	Parapenaeopsis hungerfordi
NC_038117.1	Palaemon annandalei
NC_039112.1	Munida isos
NC_039153.1	Aristeomorpha foliacea
NC_039179.1	Metapenaeus affinis
NC_039373.1	Palaemon capensis
NC_039671.1	Panulirus argus
NC_039847.1	Octopus cyanea
NC_039848.1	Callistoctopus luteus
NC_039964.1	Pleoticus muelleri
NC_040124.1	Portunus gracilimanus
NC_040140.1	Penaeus latisulcatus
NC_040855.1	Heterocarpus spp. (same barcode for AY_612880.1 Heterocarpus parvispina, MK_470779.1 Heterocarpus hayashii and NC_040855.1 Heterocarpus ensifer)
NC_040987.1	Euphausia superba
NC_041153.1	Ibacus alticrenatus
NC_041155.1	Puerulus angulatus
NC_042173.1	Metapenaeus joyneri
NC_042240.1	Paralithodes platypus
NC_042695.1	Ovalipes punctatus
NC_044093.1	Octopus spp. (same barcode for NC_044093.1 Octopus mimus and KF_373764.1 Octopus hubbsorum)
NC_044425.1	Scyllarides squammosus
NC_045090.1	Palaemon sinensis
NC_049899.1	Amphioctopus neglectus
NC_050168.1	Palaemon adspersus
NC_050266.1	Palaemon serratus
NC_050675.1	Metacarcinus magister

NC_050686.1	Taku spinosocarinatus
NC_051962.1	Cancer pagurus
NC_052010.1	Scyllarides haanii
NC_052726.1	Chionoecetes japonicus
NC_052749.1	Panulirus longipes
NC_052750.1	Panulirus penicillatus
NC_052881.1	Octopus sinensis
NC_053568.1	Lophosquilla costata
NC_053854.1	Gonodactylaceus randalli
NC_057954.1	Octopus fitchi
NC_060311.1	Gonodactylus smithii
NC_060364.1	Faughnia haani
NC_060589.1	Neogonodactylus bredini
NC_060590.1	Neogonodactylus oerstedii
NC_060621.1	Charybdis hellerii
NC_063582.1	Odontodactylus havanensis
NC_063583.1	Hemisquilla californiensis
NC_063584.1	Mesacturoides brevisquamatus
NC_063585.1	Squilla biformis
NC_063586.1	Alima pacifica
NC_065642.1	Rossia macrosoma
NC_068255.1	Penaeus duorarum
NC_068523.1	Chionoecetes spp. (same barcode for NC_068523.1 Chionoecetes bairdi and MT_335860.1 Chionoecetes opilio)
NC_069041.1	Solenocera spp. (same barcode for NC_069041.1 Solenocera melantho and NC_030280.1 Solenocera crassicornis)
NC_069188.1	Sepia orbignyana
NC_069189.1	Sepia elegans
NC_069192.1	Octopus salutii
NC_072237.1	Aristaeopsis edwardsiana
NC_081992.1	Procletes levicarina
OM_212821.1	Sepia ramani
OP_117380.1	Uroteuthis spp. (same barcode for OP_117380.1 Uroteuthis singhalensis and KF489893.1 Uroteuthis duvaucelii)
OP_851627.1	Sepia spp. (same barcode KX_984292.1 Sepia pharaonis and OP_851627.1 Sepia ramani)
WS_000001.1	Sepia officinalis
WS_000002.1	Sepia officinalis
WS_000003.1	Sepia officinalis
WS_000004.1	Octopus vulgaris
WS_000005.1	Octopus vulgaris
WS_000006.1	Octopus vulgaris
WS_000007.1	Sepia pharaonis
WS_000008.1	Sepia pharaonis
WS_000009.1	Sepia pharaonis
WS_000010.1	Uroteuthis duvaucelii

WS_000011.1	Sepia aculeata
WS_000012.1	Illex argentinus
WS_000013.1	Doryteuthis gahi
WS_000014.1	Doryteuthis pealeii
WS_000015.1	Nototodarus sloanii
WS_000016.1	Dosidicus gigas
WS_000017.1	Uroteuthis duvaucelii
WS_000018.1	Parapenaeopsis hardwickii
WS_000019.1	Metapenaeus monoceros
WS_000020.1	Acetes chinensis
WS_000021.1	Lysmata spp. (assigned to Lysmata arvoredensis with 88 % accordance (BLASTn), too low for species assignment and same assignment for species of the same genus (LCA))
WS_000022.1	Procambarus clarkii
WS_000023.1	Acetes indicus
WS_000024.1	Penaeus duorarum
WS_000025.1	Scylla olivacea
WS_000026.1	Panulirus argus
WS_000027.1	Penaeus semisulcatus
WS_000028.1	Thenus unimaculatus
WS_000029.1	Oratosquillina perpensa
WS_000030.1	Sepia ramani
WS_000031.1	Acetes japonicus
WS_000032.1	Penaeus monodon
WS_000033.1	Penaeidae (same barcode for MT_155971.1 Mierspenaeopsis hardwickii and MT_155970.1 Metapenaeus tenuipes)
WS_000034.1	Sepia pharaonis
WS_000035.1	Cancer pagurus
WS_000036.1	Acetes japonicus
WS_000037.1	Acetes spp. (assigned to Acetes indicus with 85 % accordance (BLASTn), too low for species assignment, therefore assignment on genus level)
WS_000038.1	Decapoda (assigned to Acetes indicus with 79 % accordance (BLASTn), too low for species assignment and same assignment for species of the same order (LCA))
WS_000039.1	Acetes japonicus
WS_000040.1	Aristaeopsis spp. (assigned to Aristaeopsis edwardsiana with 95 % accordance (BLASTn), but only to 91 % of the barcode. Too low for species assignment, therefore assignment on genus level)

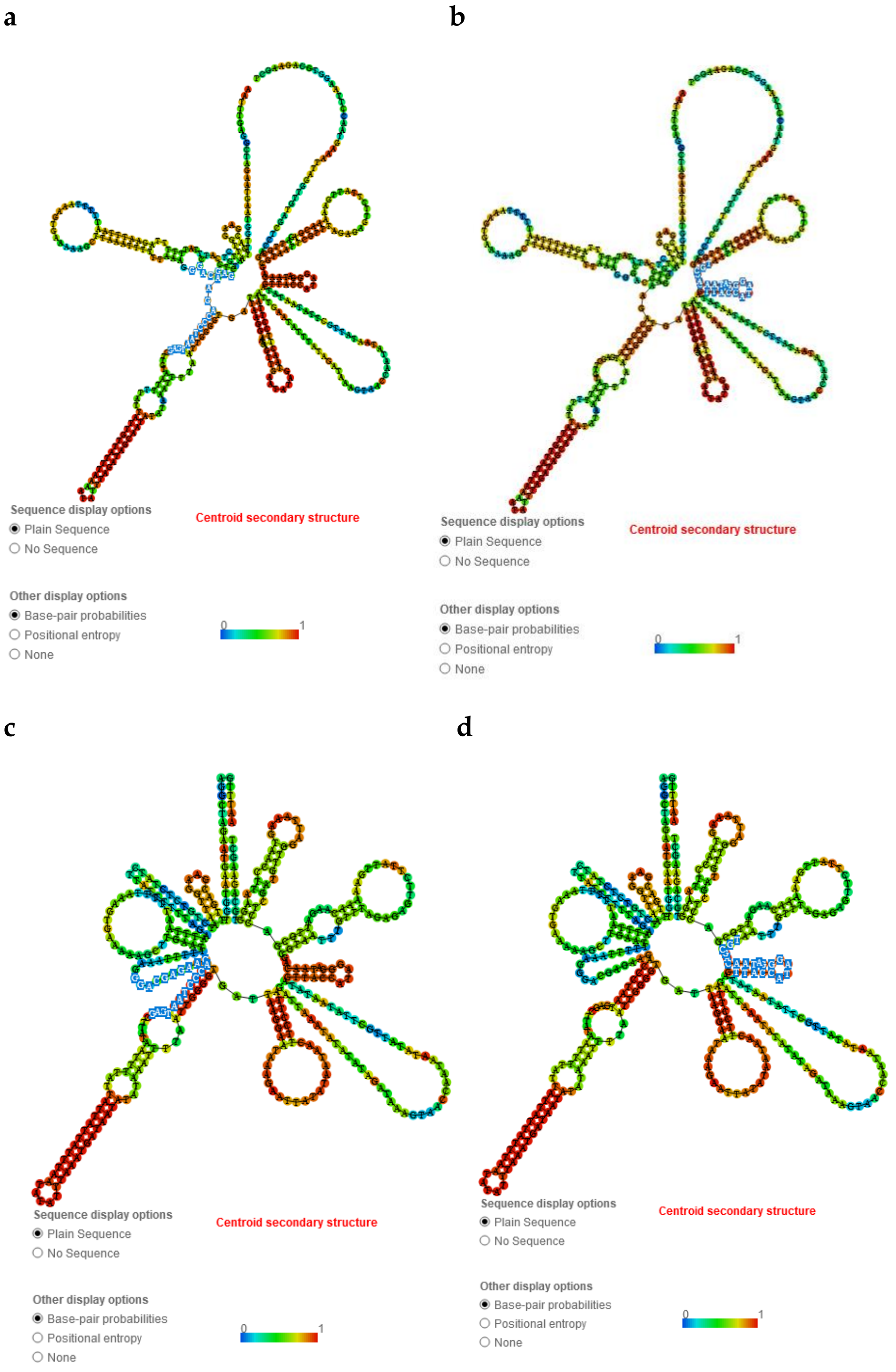

Figure S1. Comparison of predicted hairpin structures in single-stranded DNA for *Dosidicus gigas* (**a**,**b**) and *Illex illecebrosus* (**c**,**d**). Forward primer binding sites are indicated in panels **a**

and **c**; reverse primer binding sites in panels **b** and **d**. While the overall structures are similar, *D. gigas* exhibits less favorable configurations, including longer hairpin stems [62]. Nevertheless, *I. illecebrosus* was either not or minimally detected in DNA extract mixtures containing species other than *D. gigas*.

62. Fan, H.; Wang, J.; Komiyama, M.; & Liang, X. Effects of secondary structures of DNA templates on the quantification of qPCR. J. Biomol. Struct. Dyn. 2019, 37, 2867-2874, doi:10.1080/07391102.2018.1498804.
